# Brain and Pituitary Transcriptome Analyses Reveal the Differential Regulation of Reproduction-Related LncRNAs and mRNAs in *Cynoglossus semilaevis*

**DOI:** 10.3389/fgene.2021.802953

**Published:** 2021-12-09

**Authors:** Yani Dong, Likang Lyu, Haishen Wen, Bao Shi

**Affiliations:** ^1^ Key Laboratory of Sustainable Development of Marine Fisheries, Ministry of Agriculture and Rural Affairs, Yellow Sea Fisheries Research Institute, Chinese Academy of Fishery Sciences, Qingdao, China; ^2^ Key Laboratory of Mariculture (Ocean University of China), Ministry of Education, Ocean Unversity of China, Qingdao, China; ^3^ Laboratory for Marine Fisheries and Food Production Processes, Pilot National Laboratory for Marine Science and Technology (Qingdao), Qingdao, China

**Keywords:** lncRNA, mRNA, brain, pituitary, reproduction, integrated analysis, transcriptome, *Cynoglossus semilaevis*

## Abstract

Long noncoding RNAs (lncRNAs) have been identified to be involved in half-smooth tongue sole (*Cynoglossus semilaevis*) reproduction. However, studies of their roles in reproduction have focused mainly on the ovary, and their expression patterns and potential roles in the brain and pituitary are unclear. Thus, to explore the mRNAs and lncRNAs that are closely associated with reproduction in the brain and pituitary, we collected tongue sole brain and pituitary tissues at three stages for RNA sequencing (RNA-seq), the 5,135 and 5,630 differentially expressed (DE) mRNAs and 378 and 532 DE lncRNAs were identified in the brain and pituitary, respectively. The RNA-seq results were verified by RT-qPCR. Moreover, enrichment analyses were performed to analyze the functions of DE mRNAs and lncRNAs. Interestingly, their involvement in pathways related to metabolism, signal transduction and endocrine signaling was revealed. LncRNA-target gene interaction networks were constructed based on *antisense*, *cis* and *trans* regulatory mechanisms. Moreover, we constructed competing endogenous RNA (ceRNA) networks. In summary, this study provides mRNA and lncRNA expression profiles in the brain and pituitary to understand the molecular mechanisms regulating tongue sole reproduction.

## Introduction

Half-smooth tongue sole is one of the most economically important marine flatfish that is widely cultured in China. However, its reproductive dysfunctions and 3 years required for sexual maturation (Shi et al., 2016; Song et al., 2020) restrict the development of tongue sole aquaculture industry, which influences the economic benefit. Therefore, understanding of the molecular mechanism of reproduction in this species is scientifically and commercially necessary.

Reproduction is a crucial function of the organism and is controlled by complex interactions between the hypothalamus, pituitary and gonads, i.e., the hypothalamic-pituitary-gonadal (HPG) axis (Abreu and Kaiser, 2016). Within this axis, the hypothalamus releases the gonadotrophin releasing hormone (GnRH), which can act on pituitary gonadotropes, triggering the release of follicle-stimulating hormones (FSH) and luteinizing hormones (LH). FSH and LH bind to their respective receptors in the ovary, leading to secretion of the sex steroid hormones, estrogen (E_2_), which stimulates vitellogenesis, and progesterone (P4), which stimulates oocyte meiosis, follicular maturation and ovulation (Yaron and Levavi-Sivan, 2011). Although the importance of the HPG axis for reproduction has been suggested, in contrast to the wealth of knowledge in mammals, the integrated molecular mechanism of HPG axis regulation in nonmammalian species has not been elucidated.

With the rapid development of RNA sequencing (RNA-Seq), the function of noncoding RNAs (ncRNAs), which were originally considered “junk RNAs” of the mammalian genome, has received increasing attention (Kopp and Mendell, 2018). Long noncoding RNAs (lncRNAs) are a group of transcripts that are longer than 200 nucleotides, without apparent protein-coding role (Quinn and Chang, 2016) and, similar to mRNAs, often contain a poly(A) tail and can be spliced (Hombach and Kretz, 2016). LncRNAs have been reported to play important roles in diverse biological processes, such as signal transduction (Arun et al., 2012), cell differentiation and development (Fatica and Bozzoni, 2014), ontogeny (Abdelmohsen et al., 2013) and immune responses (Chen et al., 2017), through transcriptional and posttranscriptional mechanisms (Dykes and Emanueli, 2017) that regulate gene expression, such as epigenetic modification (Mercer and Mattick, 2013), gene imprinting (Williamson et al., 2011), miRNA sponging (Faghihi et al., 2010) and chromatin remodeling (Tsai et al., 2010). Some studies have shown that lncRNAs are involved in the nervous system function and neurological diseases in humans (Qureshi et al., 2010; Oe et al., 2019). In addition, emerging evidence indicates that lncRNAs are involved in reproduction-related processes, including sex hormone responses (Yang et al., 2013), oocyte meiosis (Yang et al., 2020), and ovulation (Lian et al., 2020), although these studies focused mainly on humans and other mammals.

Recently, an increasing number of studies on lncRNAs have been reported in teleosts, focusing on processes such as growth and development (Ali et al., 2018; Wu et al., 2020), stress responses (Dettleff et al., 2020; Quan et al., 2020), immune responses (Liu et al., 2019; Zheng et al., 2021) and sex differentiation (Cai et al., 2019; Feng et al., 2020). However, the related knowledge still lags far behind that in mammals. Our previous research has provided lncRNA expression profiles of the tongue sole ovary, and we investigated the potential role of lncRNAs in the ovary in reproduction (Dong et al., 2021). However, in the HPG axis, the brain and pituitary are also crucial tissues in reproductive regulation. The hypothalamus, an area of the brain, can released a series of hormones that act on the pituitary, which is not only regulated by the hypothalamus but also influences the ovarian function through hormones and other regulatory factors, thus playing a connecting role in the HPG axis. However, lncRNAs in the HPG axis of tongue sole, particularly in brain and pituitary, that regulate reproduction in this fish have not been systematically analyzed.

Thus, it is necessary to identify and characterize mRNAs and lncRNAs in the reproductive neuroendocrine system to investigate their potential function in ovarian development, maturation and ovulation in tongue sole. Therefore, in this study, the brain and pituitary of tongue sole in three stages of ovarian development [stage IV: late vitellogenesis, stage V: maturation and stage VI: after ovulation (Shi et al., 2016)] were collected for sequencing to get their transcriptome profiles and explore the effect of the brain and pituitary on transcriptomic mechanisms controlling reproduction in tongue sole. This research expands the mRNA and lncRNA catalog in the tongue sole brain and pituitary and preliminarily elucidates the molecular mechanisms in which key lncRNAs and mRNAs are involved. Furthermore, our findings reveal candidate regulators for improving the efficiency of tongue sole reproduction.

## Materials and Methods

### Sample Preparation and Ethics Statement

We previously explained the feeding and sampling process of experimental fish (Dong et al., 2021). In brief, we collected triplicate brain and pituitary samples from tongue sole in three stages of ovarian development (stages IV, V and VI). The identification of ovarian developmental stages has been described in detailed in our previous studies (Dong et al., 2021). Tissues were immediately frozen in liquid nitrogen and stored at −80°C to prevent degradation before total RNA isolation.

The acquisition of the fish was approved by the Animal Care and Use Committee of the Chinese Academy of Fishery Sciences, and all experiments were conducted at the Chinese Academy of Fishery Sciences in accordance with the Guidelines for the Care and Use of Laboratory Animals at the Chinese Academy of Fishery Sciences, China.

### RNA Isolation, Library Preparation and RNA Sequencing

According to the manufacturer’s procedure, total RNA was isolated using a TRIzol reagent kit (Invitrogen, Carlsbad, CA, United States). The quality of the RNA was measured via an Agilent 2100 Bioanalyzer (Agilent Technologies, Palo Alto, CA, United States) and RNase free agarose gel electrophoresis. The rRNAs were detached from the total RNA with a Ribo-Zero^TM^ Magnetic Kit (Epicentre, Madison, WI, United States) to retain mRNAs and ncRNAs. After rRNA detached, the enriched mRNA and ncRNAs were broken into short pieces by using fragmentation buffer and were then reverse transcribed into cDNA with random primers from an RNA Library Prep Kit (NEB, United States). Before the end repair, poly (A) addition, and Illumina sequencing adapter ligation, QIAquick PCR Extraction Kit (Qiagen, Venlo, Netherlands) was used to purify the cDNA fragments after second-strand cDNA synthesis. After the digestion of second-strand cDNA with uracil-N-glycosylase (UNG), the products were filtered by agarose gel electrophoresis, PCR amplification and sequenced on the Illumina HiSeq^TM^ 4000 platform by Gene Denovo Biotechnology Co., Ltd. (Guangzhou, China).

### Sequencing Data Processing and Analysis

Raw reads were produced by sequencing and filtered with fastp (version 0.18.0) (Chen et al., 2018) removing low-quality reads. Short reads were mapped to the rRNA database with Bowtie2 (version 2.2.8) (Langmead and Salzberg, 2012). After removing mapped rRNA reads, the remaining clean reads were further aligned to the tongue sole reference genome (Chen et al., 2014) by using TopHat2 (version 2.1.1). Rebuilding and identification of transcripts was conducted with Cufflinks (v2.1.1), TopHat2 (Kim et al., 2013), Cuffmerge (Trapnell et al., 2012), and Cuffcompare. We used the Coding-Non-Coding-Index (CNCI, version 2) (Sun et al., 2013), Coding Potential Calculator (CPC, http://cpc.cbi.pku.edu.cn/) (Kong et al., 2007) and the SwissProt protein database to distinguish the protein-coding capability of the new transcripts. The intersections of the identified transcripts without protein-coding potential were described as the collection of lncRNAs, which could be classified to 5 categories: intergenic lncRNAs, bidirectional lncRNAs, intronic lncRNAs, antisense lncRNAs, and sense-overlapping lncRNAs.

### Differentially Expressed Transcripts and Functional Enrichment Analysis

We used per kilobase of transcript per million mapped reads (FPKM) to measure and normalize the expression levels of both mRNAs and lncRNAs. RSEM software is used to calculate the FPKM (Li and Dewey, 2011). The edgeR package (http://www.r-project.org/) was used to identify significant differentially expressed (DE) transcripts among the groups. We identified DE mRNA and DE lncRNA with absolute value of log_2_(Fold change) > 1 and a false discovery rate (FDR) < 0.05, and DE miRNA with absolute value of log_2_(Fold change) > 1 and *p*-value < 0.05. Gene Ontology (GO, https://geneontology.org) and Kyoto Encyclopedia of Genes and Genomes (KEGG, https://www.genome.jp/kegg/) analyses were carried out to have an insight to the biological function of the DE mRNAs. Both the GO and KEGG analyses were guided by Gene Denovo Biotechnology Co., Ltd (Guangzhou, China). GO terms and KEGG pathways with a *p*-value < 0.05 were considered significantly enriched.

### Gene Set Enrichment Analysis

We conducted Gene Set Enrichment Analysis using GSEA software from the Broad Institute (http://www.broadinstitute.org/gsea/index.jsp) and MSigDB to identify whether a set of genes in specific GO terms\KEGG pathways\Disease Ontology (DO) terms showed a significant difference between the two groups. In brief, we input a gene expression matrix and classed genes by their signal-to-noise ratios. Enrichment scores and *p*-value were calculated with default parameters, *p*-value, *p* < 0.05; *q*-value, *q* < 0.25; and normalized enrichment score (| NES|) > 1.

### LncRNA-mRNA Association Analysis

We performed *antisense* lncRNA, *cis* regulation and *trans* regulation analyses to predict the target genes of lncRNAs. Some lncRNAs may have capacities to set gene silencing, modulating transcription and impact mRNA stability. We recognize these lncRNAs as *antisense* lncRNAs. The relationships of *antisense* lncRNA and its target genes were predicted by RNAplex software (http://www.tbi.univie.ac.at/RNA/RNAplex.1.html). Some lncRNAs can regulate their nearby genes in same allele, which situated less than 10 kilobases upstream or downstream of genes. We recognize these lncRNAs as *cis* lncRNAs. Besides, some lncRNAs can act on genes located distantly. *Trans* role is lncRNA’s regulation for expression of *trans* chromosome genes. The correlation expression of lncRNAs and genes was conducted to identify the target genes of lncRNAs with *p* ≥ 0.9. Cytoscape software (v3.6.0) was used to establish and visualize the interaction network.

### Competing Endogenous RNA Network Construction

Based on the ceRNA hypothesis, we constructed mRNA-miRNA-lncRNA networks. The negatively co-expressed mRNA-miRNA pairs and lncRNA-miRNA pairs were assessed by the Spearman rank correlation coefficient (SCC), with a SCC < -0.7. The co-expressed lncRNA-mRNA pairs were assessed using the Pearson correlation coefficient (PCC) with a PCC >0.9. The significance correlations between the common miRNA sponges and the two genes were calculated using the method of hypergeometric cumulative distribution. The mRNA-miRNA-lncRNA pairs were filtered with a *p*-value < 0.05. The interaction networks were visualized using Cytoscape software (v3.6.0).

### Real-Time Quantitative PCR Validation

Sixteen transcripts‒eight mRNAs and eight lncRNAs‒were randomly selected to validate the RNA-Seq data by RT-qPCR. Primers as shown in Supplementary Table S1 were designed with Primer 5 software (Premier Biosoft International) and synthesized by Sangon biotech (Shanghai, China). RNA was reverse transcribed to cDNA using HiScript III RT SuperMix for qPCR (+gDNA wiper) (Vazyme Biotech, China). The RT-qPCR reaction system was mixed with cDNA template, primers, ChamQ SYBR Color qPCR Master Mix (Vazyme Biotech, China) and RNase-free water. All reactions were contained in 96 well plate and then conducted in a StepOne Plus Real-Time PCR system (Applied Biosystems, United States) with triplicate. Melting curves analysis was used to re-confrm the amplifcation of only a single PCR product. Using beta-2-microglobulin (*β*-2-m) as a reference, the relative expression levels of mRNA and lncRNA were quantified with the 2^−ΔΔCt^ method (Livak and Schmittgen, 2001).

## Results

### Overview of the RNA-Sequencing

In this study, 18 cDNA libraries were constructed for sequencing to conduct a mechanistic investigation of the brain and pituitary transcriptome mechanism and analyse the potential role of lncRNAs regulating the reproduction in tongue sole. A total of 734.6 million (734,595,534) and 720.1 million (720,070,248) clean reads were generated from the brain and pituitary. After removing low-quality reads, an average of 98.66 and 98.54% of the high-quality reads from the brain and pituitary, respectively, were unmapped to the rRNA database. And the rRNA mapped reads were deleted. Among the unmapped reads, the mapping ratios and unique mapping ratio of tongue sole reference genome were 76.98 and 75.97%, respectively, in the brain and 74.36 and 72.22%, respectively, in the pituitary (Supplementary Table S2).

### Functional Annotation and Classification of the Assembled Sequences

**FIGURE 1:**
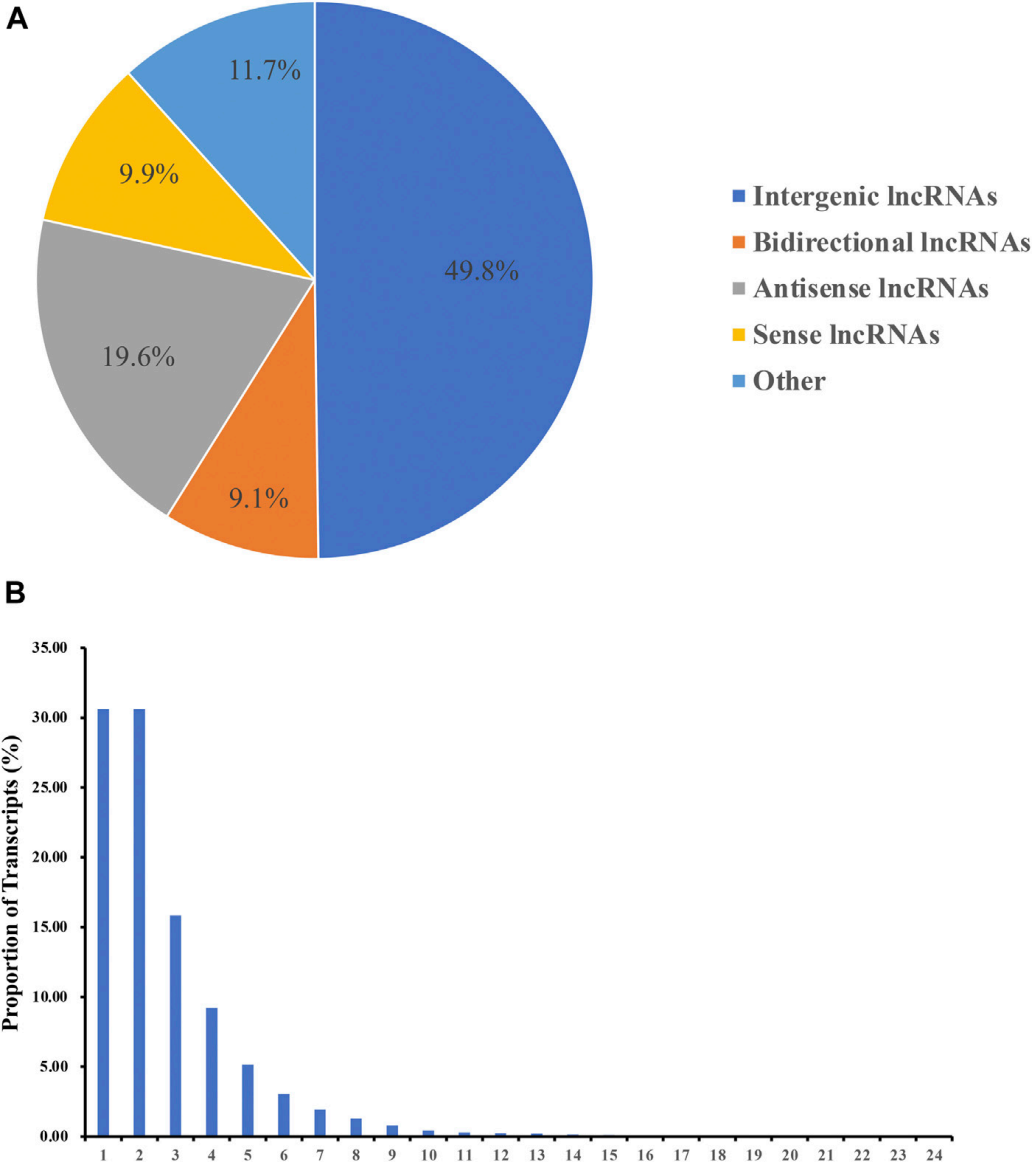
Identification of lncRNAs. **(A)** Categories of the novel lncRNA transcripts. **(B)** Number of exons in the novel lncRNA transcripts.

The novel transcripts were identified based on the location of the assembled transcripts in the reference genome, a transcript length of ≥200 bp and an exon number of ≥2. The functional annotation information of the novel transcripts is illustrated in Supplementary Table S3. The intersection of the CPC/CNCI/SwissProt data was taken to screen for lncRNAs. A total of 3,897 lncRNA transcripts were obtained, including 1,941 intergenic lncRNAs (49.8%), 355 bidirectional lncRNAs (9.1%), 763 antisense lncRNAs (19.6%), 384 sense-overlapping lncRNAs (9.9%) and 455 other lncRNAs (11.7%). No intronic lncRNAs were identified (Figure 1A). In addition, most novel lncRNA transcripts mainly contained between 1 and 4 exons and the number of lncRNA transcripts decreased with the increase of the number of exons (Figure 1B).

### Analysis of DE mRNAs and LncRNAs

**FIGURE 2:**
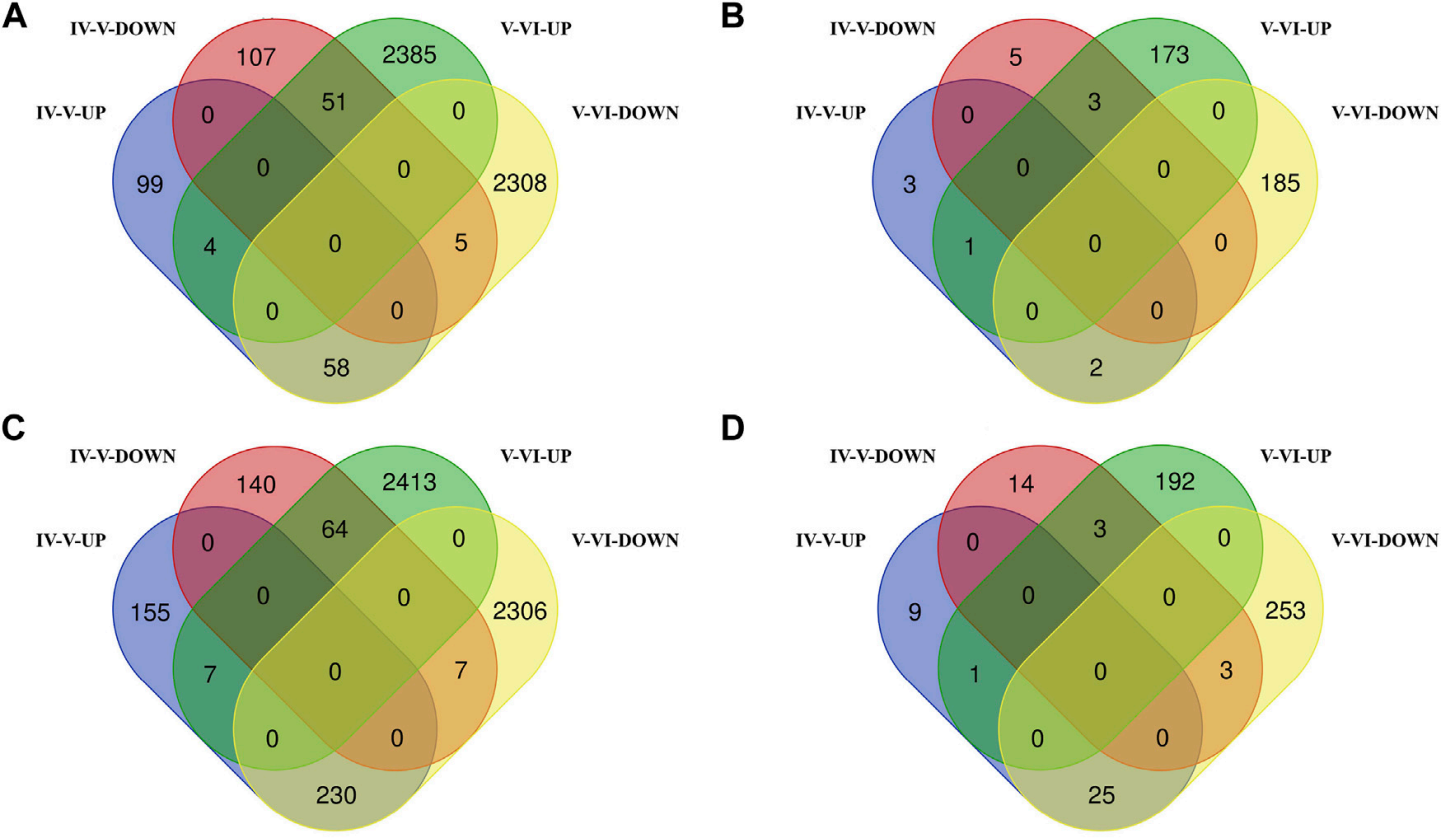
Venn diagram of DE mRNA **(A)** and lncRNA **(B)** statistics in the brain. Venn diagram of DE mRNA **(C)** and lncRNA **(D)** statistics in the pituitary.

In the brain, we identified 324 DE mRNAs, 161 upregulated and 163 downregulated, in the ovarian stages IV vs. V comparison group and identified 4,811 DE mRNAs, 2,440 upregulated and 2,371 downregulated, in the ovarian stages V vs. VI comparison group. There were 118 DE mRNAs were identified in both comparison groups (Supplementary Table S4, Figure 2A). We identified 14 DE lncRNAs, 6 upregulated and 8 downregulated, in the ovarian stages IV vs. V comparison group and identified 364 DE lncRNAs, 177 upregulated and 187 downregulated, in the ovarian stages V vs. VI comparison group. There were 6 DE lncRNAs were identified in both comparison groups (stages IV vs. V and V vs. VI) (Supplementary Table S4, Figure 2B).

In the pituitary, we identified 603 DE mRNAs, 392 upregulated and 211 downregulated, in the ovarian stages IV vs. V comparison group and identified 5,027 DE mRNAs, 2,484 upregulated and 2,543 downregulated, in the ovarian stages V vs. VI comparison group. There were 308 DE mRNAs were identified in both comparison groups (Supplementary Table S5, Figure 2C). We identified 55 DE lncRNAs, 35 upregulated and 20 downregulated, in the ovarian stages IV vs. V comparison group and identified 477 DE lncRNAs, 196 upregulated and 281 downregulated, in the ovarian stages V vs. VI comparison group. There were 32 DE lncRNAs were identified in both comparison groups (Supplementary Table S5, Figure 2D).

**FIGURE 3:**
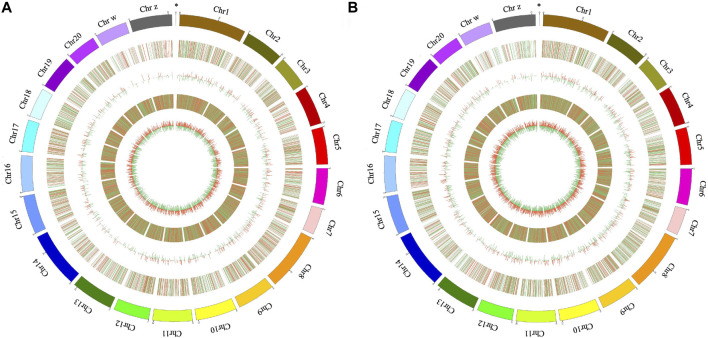
Circos plot of DE lncRNAs and mRNAs in the brain in the ovarian stages IV vs. V **(A)** and stages V vs. VI **(B)** comparison groups. The circle from outside to inside shows the autosomal distribution in tongue sole, the chromosomal locations of the DE lncRNA, the histogram of DE lncRNAs at different positions, the distribution of DE mRNAs on the chromosomes and the histogram of DE mRNAs at different positions, respectively; the red line represents upregulation, and the green line represents downregulation; higher columns indicate higher numbers of DE genes. The “*” symbol indicates the mitochondrial genome.

DE mRNAs and lncRNAs were illustrated in circos plots. The DE mRNAs and lncRNAs in the brain and pituitary in two comparison groups (stages IV vs. V and V vs. VI) are presented in Figure 3 and Supplementary Figure S1.

### GO and KEGG Pathway Enrichment Analyses of DE mRNAs

We performed GO and KEGG enrichment analyses to identify the biological functions of the DE mRNAs. As shown in Supplementary Table S6, in the ovarian stages IV vs. V comparison, the most enriched GO terms in the brain were extracellular region part and NAD(P)^+^ transhydrogenase activity, which were in the cellular component and molecular function categories, respectively. In the biological process category, the significantly enriched GO terms included cell migration, cell motility and cellular localization. In the ovarian stages V vs. VI comparison, the most enriched GO terms in the brain were cytosol and activin receptor signaling pathway, which were in the cellular component and biological process categories, respectively. As shown in Supplementary Table S6, in the ovarian stages IV vs. V comparison, the significantly enriched GO terms in the pituitary included non-membrane-bounded organelle, cytoskeleton, oxidoreductase activity, growth factor binding, carbohydrate metabolic process and so on. In the ovarian stages V vs. VI comparison, the significantly enriched GO terms in the pituitary included cytoskeleton, transport vesicle membrane, nucleoside-triphosphatase activity, oxidoreductase activity and chromatin organization. Furthermore, several GO terms related to reproduction, including female pregnancy sex chromosome and fatty acid synthase activity, were enriched.

**FIGURE 4:**
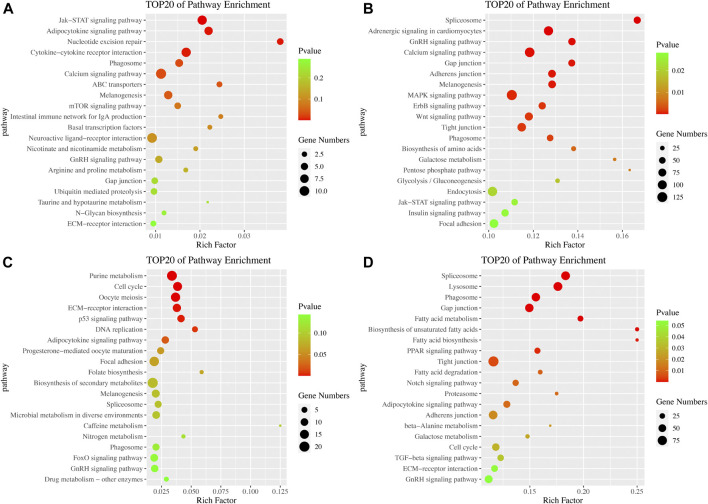
Pathway enrichment analysis of DE mRNAs. KEGG pathway enrichment in the brain in the ovarian stages IV vs. V **(A)** and stages V vs. VI **(B)** comparison groups. KEGG pathway enrichment in the pituitary in the ovarian stages IV vs. V **(C)** and stages V vs. VI **(D)** comparison groups.

In the KEGG pathway analysis, we established a *p*-value of 0.05 as the threshold. In the brain, 8 and 23 pathways were significantly enriched in the ovarian stages IV vs. V and stages V vs. VI comparison groups, respectively. Among these pathways, the Jak-STAT signaling, calcium signaling and melanogenesis pathways were significantly enriched in both comparison groups (Figure 4A). In the ovarian stages IV vs. V comparison, the cytokine-cytokine receptor interaction pathway was significantly enriched. In the ovarian stages V vs. VI comparison, several signaling pathways, including the GnRH signaling pathway, MAPK signaling pathway, ErbB signaling pathway, Wnt signaling pathway and insulin signaling pathway, were identified (Figure 4B). In the pituitary, 7 and 18 pathways were significantly enriched in the ovarian stages IV vs. V and stages V vs. VI comparison groups, respectively. Among these pathways, the adipocytokine signaling pathway and cell cycle pathway were significantly enriched in both comparison groups. In the ovarian stages IV vs. V comparison, pathways associated with reproduction and the cell cycle, such as oocyte meiosis, ECM-receptor interaction and p53 signaling pathway, were identified (Figure 4C). In the ovarian stages V vs. VI comparison, several pathways related to metabolism, including fatty acid biosynthesis, degradation and metabolism, were identified (Figure 4D).

### GSEA

We carried out GSEA to identify more potential pathways that are not significantly different but have important biological significances. Generally, the significance of gene sets was established based on the following criteria: absolute normalized enrichment score (|NES|)>1, nominal (NOM) *p*-value < 0.05, and FDR *q*-value < 0.25 (Supplementary Table S7). In the brain, GSEA showed that 6 and 4 pathways were significantly enriched in the ovarian stages IV vs. V and stages V vs. VI comparisons, respectively. Among these pathways, some were related to genetic information processing, such as proteasome, protein export and ribosome biogenesis in eukaryotes. In addition, some pathways were related to immunity, such as intestinal immune network for IgA production and Toll-like receptor signaling pathway. In the pituitary, GSEA showed that 27 and 12 pathways were significantly enriched in the ovarian stages IV vs. V and V vs. VI comparisons, respectively. Some pathways, such as the p53 signaling pathway, cell cycle, glycosphingolipid biosynthesis–lacto and neolacto series, NOD-like receptor signaling pathway, DNA replication and Toll-like receptor signaling pathway, were significantly enriched in both comparison groups.

### Identification and Enrichment Analysis of LncRNA Target Genes

**FIGURE 5:**
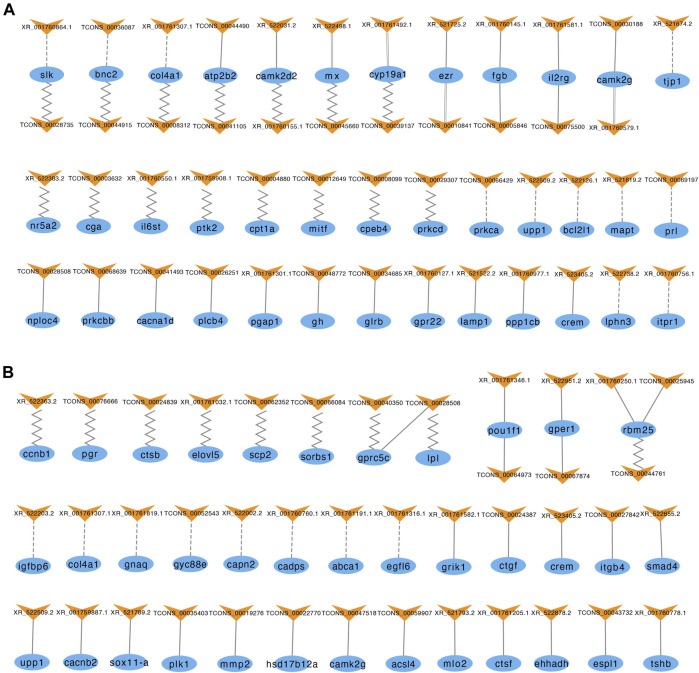
LncRNAs and their *antisense*, *cis*, and *trans* target genes in the brain **(A)** and pituitary **(B)**. The blue ellipses represent mRNAs, and the yellow “V” symbols represent lncRNAs. The dashed, solid, parallel and jagged lines indicate interactions between DE lncRNAs and their *antisense*, *cis*, and *trans positive* and *negative* target genes.

To further predict the potential roles of the lncRNAs involved in modulating the reproductive process, we constructed interaction networks of lncRNAs and their *antisense*, *cis* and *trans* target genes (Figure 5). In the brain, 57 lncRNAs had the potential to regulate 40 of the genes listed in Supplementary Table S8. Among these lncRNAs, 11 exhibited *antisense* complementary correlations with 12 DE mRNAs, 19 showed potential *cis* target regulatory relationships with 16 DE mRNAs, and 19 lncRNA-mRNA coexpression pairs were identified. In the pituitary, 42 lncRNAs had the potential to regulate 38 of the genes listed in Supplementary Table S8. Among these lncRNAs, 9 exhibited *antisense* complementary correlations with 12 DE mRNAs, 26 showed potential *cis* target regulatory relationships with 23 DE mRNAs, and 9 lncRNA-mRNA coexpression pairs were identified.

The top 20 KEGG pathways of the three types of regulatory relationships are shown in Supplementary Figure S2 (also see Supplementary Table S9). The target genes of these DE lncRNAs were enriched in numerous KEGG pathways, including some signal transduction pathways, such as TGF-beta signaling pathway, Wnt signaling pathway and ErbB signaling pathway; some signaling molecule and interaction pathways, such as cytokine-cytokine receptor interaction, neuroactive ligand-receptor interaction, ECM-receptor interaction and cell adhesion molecules (CAMs); and some pathways related to cell growth and death, such as apoptosis, cell cycle, fatty acid biosynthesis and steroid hormone biosynthesis pathways. These pathways directly or indirectly influence reproductive processes, indicating that these lncRNAs may play a role in reproduction.

### mRNA-MiRNA-LncRNA Interaction Analysis

We used MIREAP, miRanda and TargetScan to predict the targets of each miRNA after DE mRNAs, lncRNAs and miRNAs were recognized. CeRNA networks were built based on the mRNA-miRNA and lncRNA-miRNA pairs. In these ceRNA networks, each pair of mRNAs and lncRNAs were targeted and negatively co-expressed with a joint miRNA (Supplementary Table S10).

**FIGURE 6:**
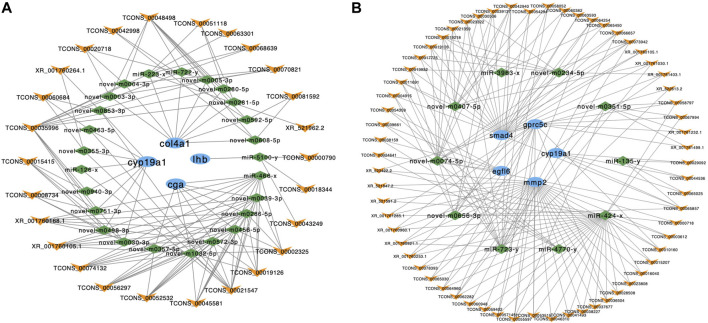
The ceRNA interaction networks in the brain and pituitary. **(A)** The ceRNA network of *cyp19a1*, *col4a1*, *lhb* and *cga* in the brain. **(B)** The ceRNA network of *smad4*, *gpcr5c*, *cyp19a1*, *mmp2* and *egfl6* in the pituitary. The blue ellipses represent mRNAs, the green diamonds represent miRNAs, and the yellow “V” symbols represent lncRNAs.

According to the result of ceRNA network and pathway enrichment analysis, we selected 4 reproduction-related genes in the brain‒*cga*, *lhb*, *cyp19a1* and collagen alpha-1 (IV) chain (*col4a1*) ‒to predict their interactions with lncRNAs. A total of 26 lncRNAs interrelated with these 4 mRNAs through competitive binding of 25 miRNAs. We also selected 5 reproduction-related genes in the pituitary‒*cyp19a1*, *mmp2*, *egfl6*, *smad4* and *gprc5c*‒to predict their interactions with lncRNAs. A total of 63 lncRNAs interrelated with these 5 mRNAs through competitive binding of 10 miRNAs (Figure 6).

GO and KEGG enrichment analyses were conducted to analyze the functions of the mRNAs in the ceRNA networks. GO terms and KEGG pathways with *p*-value < 0.05 were regarded as significant enrichment. In the brain, overall, 99 GO terms were identified. In the pituitary, overall, 132 GO terms were identified (Supplementary Table S11). In addition, brain mRNAs involved in ceRNA network were annotated to 9 pathways, including MAPK signaling pathway and endocytosis. In pituitary, mRNAs involved in ceRNA network were annotated to 13 pathways, including reproduction-related pathways such as MAPK signaling pathway, fatty acid elongation, oocyte meiosis and progesterone-mediated oocyte maturation (Supplementary Figure S3).

### Validation of RNA-Seq Data by RT-qPCR

**FIGURE 7:**
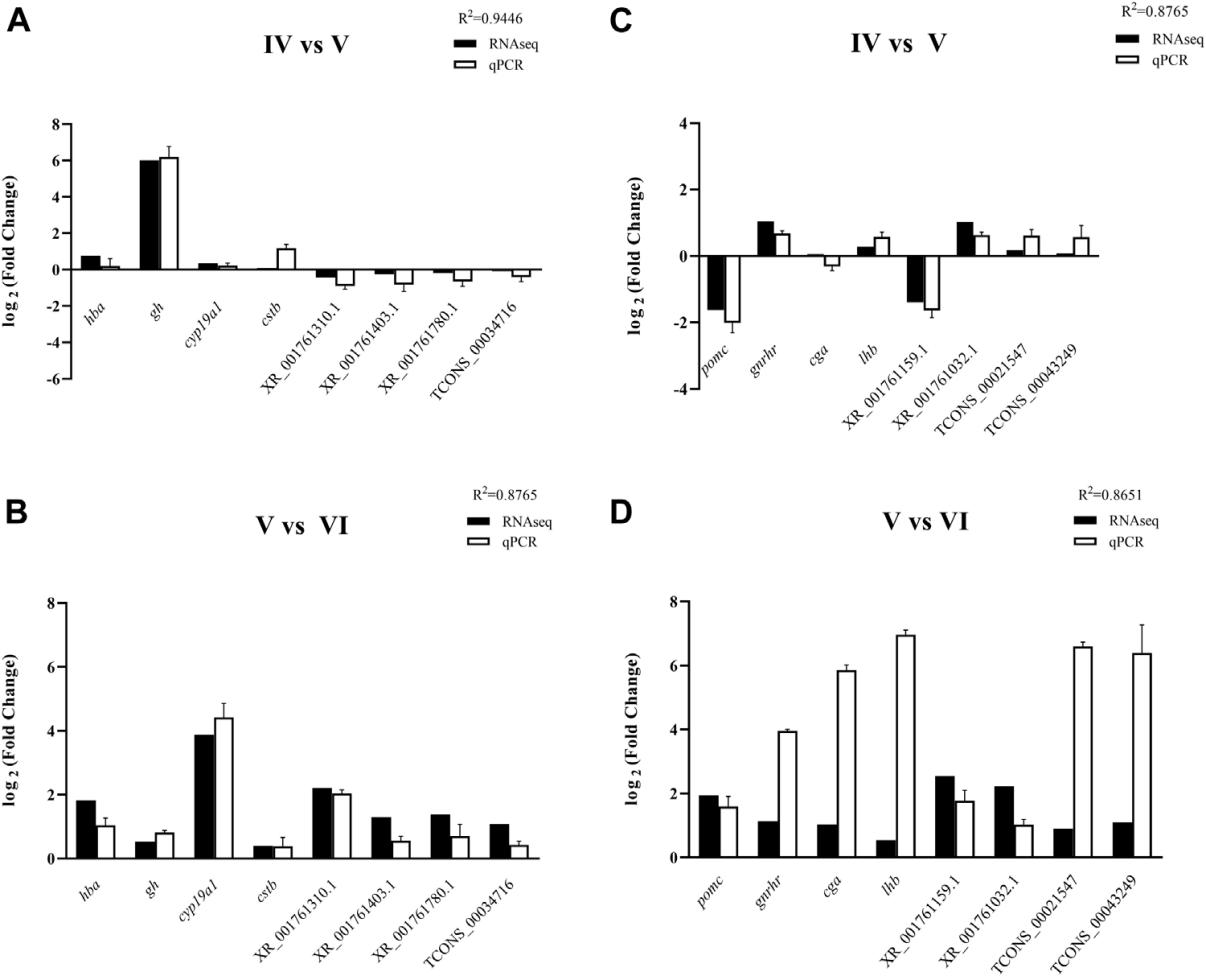
RT-qPCR validation of 4 DE genes and 4 DE lncRNAs in the brain of tongue sole in ovarian stages IV vs. V **(A)** and stages V vs. VI **(B)** and in the pituitary in ovarian stages IV vs. V **(C)** and stages V vs. VI **(D)**. The expression levels of the selected genes were normalized to the β-2-m gene.

An equal number (8) of DE mRNAs and DE lncRNAs were selected to evaluate the results of RNA-Seq. Their expression levels in the brain and pituitary from the ovarian stages IV vs. V and V vs. VI comparison groups were quantified by RT-qPCR. The expression patterns of selected transcripts from the RNA-Seq analysis were similar with RT-qPCR experimental validation results. These findings showed that our RNA-Seq results were usability and accuracy (Figure 7).

## Discussion

Reproduction, a complex biological process involving many organs, is precisely controlled by the HPG axis in vertebrates. The hypothalamus and pituitary can secrete a series of hormones triggering ovarian development, maturation and ovulation to maintain the reproductive cycle. However, more recent studies of the role of lncRNAs in reproduction in tongue sole have focused mainly on the ovary (Dong et al., 2021). In this study, the transcriptome analysis of the brain and pituitary of tongue sole at three ovarian developmental stages was performed to investigate the key gene functions and the relationships of mRNAs and lncRNAs. We conducted KEGG pathway analysis to identify the biological functions of the DE genes. In this study, some pathways were significantly enriched in the brain at ovarian stages V vs. VI, for example, GnRH signaling pathway, calcium signaling pathway and MAPK signaling pathway. Previous studies have shown that the principal molecule controlling the HPG axis in mammals is the decapeptide GnRH, which is secreted by the hypothalamus and is a major regulator of reproduction. GnRH acts on the pituitary where its receptors located to control the synthesis and release of the gonadotropins, LH and FSH (Blanco, 2020). These hormones are glycoprotein heterodimers, which are composed of a common α submit (Cga, or αGSU) and unique β subunits (Lhβ and Fshβ) (Shi et al., 2015; Coss, 2018).

**FIGURE 8:**
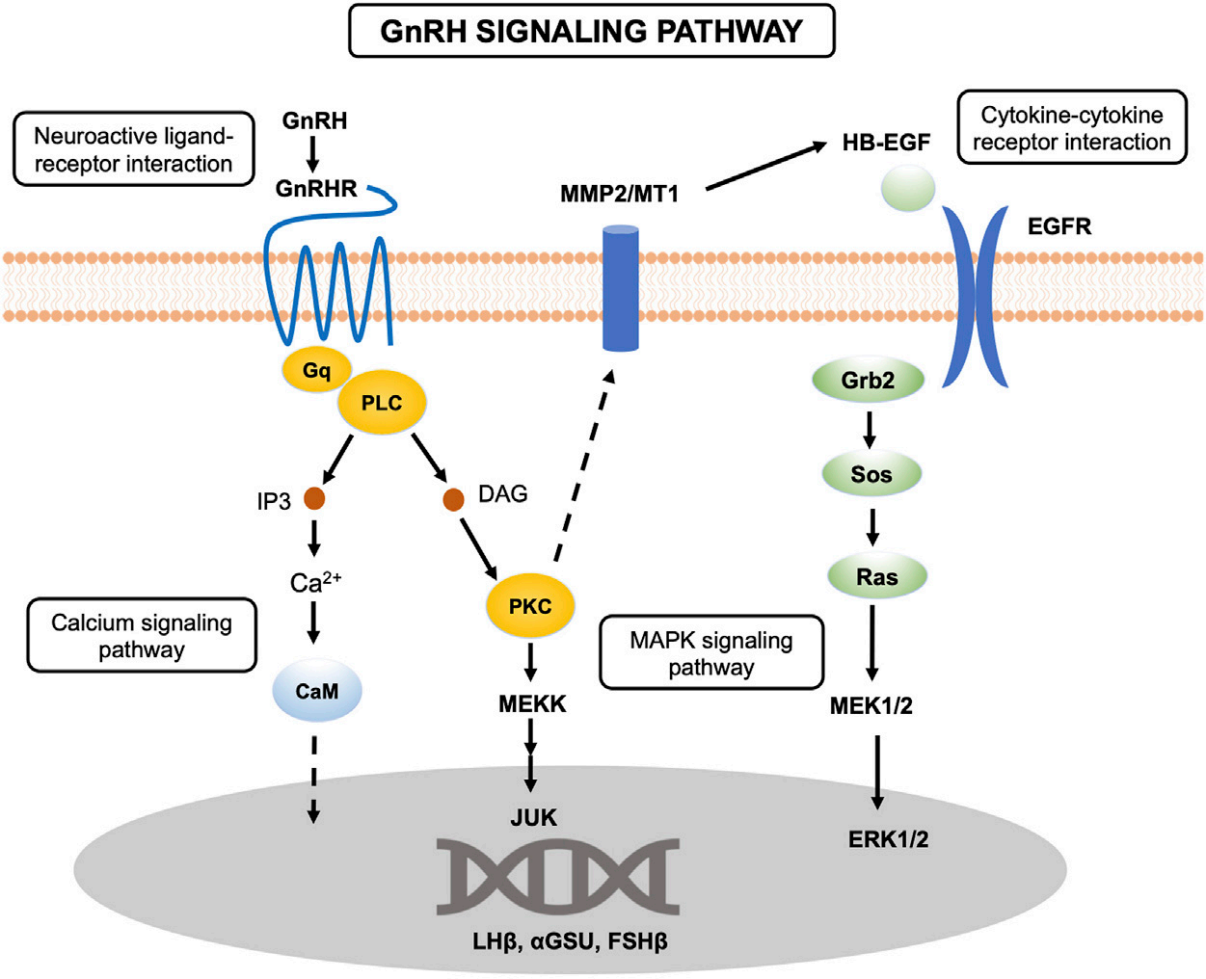
Schematic overview of the GnRH signaling pathway.

As shown in Figure 8, the GnRHR, which belongs to the 7 transmembrane domain-containing class A superfamily of G-protein-coupled receptors (GPCRs), binds to G_q/11_ proteins to activate phospholipase C (PLC), which conveys its signal to diacylglycerol (DAG) and inositol 1,4,5-trisphosphate (IP3) (Chang and Pemberton, 2018; Janjic et al., 2019). In this study, we found that members of the GPCR family, including GPCR family C group 5 member C (Gprc5c) *GPR52*, and *GPR4*, were significantly differentially expressed in the pituitary at ovarian stages V vs. VI. All of these genes were expressed mainly in the brain and pituitary. DAG and IP3 activate the intracellular protein kinase C (PKC) pathway and stimulate the release of intracellular calcium, respectively. The activation of PKC transactive the epidermal growth factor (EGF) receptor and active mitogen-activated protein kinases (MAPKs). Active MAPKs translocation to the nucleus leads to activation of transcription factors and rapid induction of early genes, including *lhβ*, *fshβ* and *cga*. In the present study, the *cga* was highly expressed in the pituitary compared to the ovary and brain. In addition, *cga* was significantly differentially expressed in the brain in the ovarian stages IV vs. V comparison and exhibited the highest expression in the brain during ovarian stage IV. Similar to *cga*, *lhβ* was also expressed mainly in the pituitary and brain, especially in the pituitary. The *lhβ* was significantly differentially expressed in the brain in the ovarian stages IV vs. V comparison and exhibited the highest expression in the brain during ovarian stage IV.

In the present study, we found that the cytokine-cytokine receptor interaction pathway was significantly enriched in the brain at ovarian stages IV vs. V. Cytokines are soluble extracellular proteins or glycoproteins that are crucial intercellular regulators of cells involved in innate immunity, cell growth, cell differentiation, and cell death. Cytokines are released by diverse cells in the body, and they stimulate responses by binding to specific receptors on the surface of target cells. As shown in the GnRH signaling pathway (Figure 8), EGF, as a kind of cytokine, binds to EGFR on the cell surface to activate downstream pathways. In vertebrates, hypothalamic peptides affect the production of pituitary hormones, which are regulated by several growth factors, including EGF, one of the most extensively studied growth factors (Smith et al., 2015). In mammals, EGF can induce prolactin (PRL) and LH secretion, suggesting that EGF might play a crucial role in growth and reproduction (Przylipiak et al., 1988). A previous study in zebrafish (*Danio rerio*) showed that EGF did not affect *lhβ* expression in pituitary cells (Lin and Ge, 2009). However, EGF was found to significantly reduce *lhβ* expression in grass carp (*Ctenopharyngodon idellus*) (Hu et al., 2020). In our study, epidermal growth factor-like protein 6 isoform X2 (*egfl6*) was found to be significantly differentially expressed at ovarian stages V vs. VI and exhibited the highest expression during ovarian stage IV. Moreover, *lhβ* exhibited the highest expression during ovarian stage IV in the pituitary, with the same expression trend as *egfl6*, indicating that EGF may also affect pituitary hormones in tongue sole. Furthermore, we found that epidermal growth factor receptor isoform X2 (*egfr*) was significantly differentially expressed in the brain at ovarian stages V vs. VI.

Metabolism has a strictly connection with reproduction, and many studies have focused on elucidating the mechanism by which signals from the periphery are transmitted to the HPG axis in multiple metabolic states (Sliwowska et al., 2014). Maintaining the balance between reproduction and energy metabolism is important. The process of reproduction requires energy, which is stored mainly as fat, glycogen and glucose. Insulin has been recognized as a key hormone for metabolic regulation in vertebrates (Irwin, 2019). The major function of insulin is to maintain peripheral glucose homeostasis through stimulation of glucose uptake, oxidation and storage (Deck et al., 2017). However, insulin also plays an important role in regulating reproduction and may play a vital role in the interaction between metabolism and reproduction in mammals (Sliwowska et al., 2014). Studies have shown that twice daily insulin treatment can reverse the 50% reduction in LH in diabetic male rats (*Rattus norvegicus*) compared to nondiabetic controls (Dong et al., 1991). However, research showed that in a growth-restricted, hypogonadotropic lamb model, central injection of insulin did not increase LH secretion (Hileman et al., 1993). Therefore, the role of insulin as a regulator in the HPG axis in mammals remains controversial. In present study, we found that the insulin signaling pathway was significantly enriched in the brain at ovarian stages V vs. VI, indicating that insulin may impact reproduction in tongue sole. In the silver pomfret (*Pampus argenteus*), gonadal development, especially vitellogenesis and spermatogonia proliferation, is closely related to IGFs (Gu et al., 2021). IGF has also been proven to control reproduction in the sapphire devil, *Chrysiptera cyanea* (Mahardini et al., 2018). In our study, insulin-like growth factor 2 (*igf2*) was differentially expressed in the pituitary at ovarian stages V vs. VI. Other metabolic pathways were enriched in the pituitary at ovarian stages V vs. VI, for example, fatty acid metabolism, biosynthesis and degradation and galactose metabolism. Fatty acids have already been proven to play an important role in reproduction (Sorbera et al., 2001).

The extracellular matrix (ECM) is a noncellular component of tissues and organs that maintains cell and tissue structure and functions. The ECM exerts direct or indirect control over cellular activities such as cell adhesion, migration, differentiation, proliferation and apoptosis (Walker et al., 2018). The ovary has been proven to undergo extensive tissue remodeling throughout each reproductive cycle (Curry Jr and Osteen, 2003). The matrix metalloproteinase (MMP) family is the important enzyme family accountable for ECM remodeling. The MMP system is generally believed to participate in follicle rupture during ovulation in medaka (*Oryzias latipes*) (Ogiwara et al., 2005; Takahashi et al., 2013). *Mmp2* has been observed to possibly be involved in regulating the activity of stem/progenitor cells that give rise to new granule neurons (Sîrbulescu et al., 2015). Studies have shown that the activation of extracellular signal-regulated kinases 1 and 2 (ERK1/2), which is included in response to GnRHR, occurs through the sequential activation of PKC, MMPs and EGF receptor transactivation (Shah and Catt, 2004). Further studies showed that MMP2 and MMP9 may participate in the endocrine regulation of pituitary gonadotrophs, suggesting a vital role for MMPs in GnRH signaling (Roelle et al., 2003). In addition, MMP2 induces the hydrolysis of type IV collagen, which constitutes the basement membrane, and membrane-type 2-MMP degrades type I collagen present in the theca cell layer in medaka (Ogiwara et al., 2005). Research has indicated that the *col4a1* knockdown decreases cell viability and suppresses cell cycle progression in breast cancer cells (Salem et al., 2016). Moreover, the type IV collagen has been detected in the basement membranes of the rat anterior pituitary (Vila-Porcile et al., 1992). Collagen, as a component of the ECM, participates in the tissue remodeling. We found that *mmp2* was differentially expressed and exhibited the highest expression at ovarian stage VI in the pituitary. We also found that *col4a1* was differentially expressed and exhibited the highest expression at ovarian stage V in the pituitary. In ovarian stages V vs. VI, *mmp2* exhibited increased expression, while *col4a1* exhibited decreased expression. To date, the detailed function of *mmp2* and *col4a1* in the reproduction of teleosts is poorly characterized. Considering these observations, it is tempting to speculate that *mmp2* and *col4a1* in the pituitary are associated with neuroendocrine regulation by a novel mechanism in the reproduction of tongue sole.

Recent studies have identified the roles of lncRNAs in reproduction, focusing mainly on the ovary. However, the expression patterns and potential physiological funtions of lncRNAs in the brain and pituitary are unclear. Therefore, we used transcriptome sequencing to explore lncRNA expression profiles in the brain and pituitary at three ovarian stages. A total of 22,549 lncRNAs (9,816 known and 12,733 novel) were identified. The present study indicated that brain and pituitary DE lncRNAs and their target DE genes might have regulatory roles in reproduction in tongue sole. Thus, we constructed lncRNA-mRNA interaction networks based on their *antisense* and *cis* regulatory mechanisms or coexpression relationships. The networks indicate that *smad4* is the *cis* target gene of lncRNA XR_522655.2. The porcine SMAD4 protein has high expression in granulosa cells (GCs) and oocytes from primary, preantral, and antral follicles and lower expression in apoptotic ovarian GCs, indicating that SMAD4 may participate in ovarian development and selection (Liu et al., 2014). SMAD4 is a key component of the TGF-β signaling pathway, which participated in the various processes in the mammalian ovary and participates in feedback regulation between the ovary and pituitary (Yu et al., 2013; Du et al., 2020). *Smad4* dysregulation is associated with disorders and delays in nervous system development (Liu et al., 2016). In goldfish (*Carassius auratus*), *smad4* has been proven to have a high expression in the pituitary and might affect FSH synthesis (Lau and Ge, 2005). In addition, accumulating evidence shows that several lncRNAs may have a potential role in regulating *smad4*, for example, lncRNA PCAT7 in prostate cancer cells (Lang et al., 2020), lncRNA 9884 in renal inflammatory cells (Zhang et al., 2019), LINP1 in lung cancer cells (Zhang et al., 2018) and LIN-LET in bladder cancer cells (Zhuang et al., 2017). In our study, we also found that lncRNA XR_522655.2 might have a *cis* regulatory relationship with *smad4* in the brain and pituitary of tongue sole for reproduction.

In the lncRNA-mRNA networks, the lncRNA TCONS_00069197 is *antisense*-acting toward its target gene *prl*, a gene encoding PRL. PRL is an anterior pituitary hormone with one of the broadest ranges of function among vertebrate hormones (Whittington and Wilson, 2013). PRL is a cytokine based on its structure and receptor type, which possesses 1 transmembrane domain, together with growth hormone (Dobolyi et al., 2020). In mammals, PRL has been proven to take part in reproductive development and cycling, including oocyte meiosis and maturation (Sorokowski et al., 2019). PRL also plays an important role in reproduction of nonmammalian species. In chickens, PRL participates in the mechanisms orchestrating ECM turnover during ovarian follicular development in the hen (*Gallus domesticus*) ovary by regulating the transcription, translation, and/or activity of some components of the MMP system (Hrabia et al., 2021). Similarly, in fish, PRL has been shown to play a role in reproductive development and sexual maturity. The *prl* has a significantly lower expression level in juvenile male blue gouramis (*Trichogaster trichopterus*), than in reproductively mature males, suggesting that PRL may involve in endocrine control of gonadal development and reproduction (Degani et al., 2010). Furthermore, prl has been detected in all the brain areas of orange-spotted grouper (*Epinephelus coioides*) (Zhang et al., 2004), yellow perch (*Perca flavescens*) (Lynn and Shepherd, 2007), goldfish (Imaoka et al., 2000), and rainbow trout (*Oncorhynchus mykiss*) (Yang et al., 1999). In this study, *prl* displayed high expression in the pituitary and brain, especially in stage IV. Similar to *prl*, the lncRNA TCONS_00069197 also had relatively high expression in the pituitary and brain and especially in stage IV, indicating that this lncRNA may play a role in regulating the nero- and endocrine reproductive axes, such as steroidogenesis and gonadogenesis, in tongue sole.

We also constructed mRNA-miRNA-lncRNA networks based on the ceRNA hypothesis. Interestingly, in the brain, the genes closely related to reproductive processes, including *cga*, *lhb*, *cyp19a1* and *col4a1*, were potentially modulated by 26 lncRNAs via competition for 25 miRNAs. In the pituitary, the genes closely associated with reproductive processes, including *cyp19a1*, *mmp2*, *egfl6*, *smad4* and *gprc5c*, were potentially modulated by 63 lncRNAs via competition for 10 miRNAs. Aromatase is encoded by the *cyp19a1s* gene. It is a key enzyme that converts androgens into estrogens and plays a crucial role in regulating numerous physiological functions in vertebrates, including sex differentiation, gonadal development and feedback effects of steroid hormones on gonadotropin secretion (Lin et al., 2020). In teleosts, there are two types of *cyp19a1s*, encoding aromatase a and b, which are expressed mainly in the gonads and brain, respectively. Teleost *cyp19a1a* expressed in the gonad is involved in gonadal sex differentiation to trigger and maintain ovarian differentiation (Guiguen et al., 2010). The expression of teleost *cyp19a1b* in the brain is connected with brain cell proliferation, neurogenesis and brain development and repair (Ulhaq and Kishida, 2018). In this study, *cyp19a1* was expressed mainly in the brain and pituitary. Sixteen lncRNAs were shown to modulate *cyp19a1* via 8 miRNAs in the brain, and 4 lncRNAs were shown to modulate *cyp19a1* via 1 miRNA in the pituitary. Moreover, the KEGG pathways progesterone-mediated oocyte maturation, oocyte meiosis and MAPK signaling pathway were enriched in DE mRNAs in the ceRNA network. These results indicate that lncRNAs may be regulators of gene expression in the brain and pituitary via miRNAs, which could play a potential role in tongue sole reproduction regulated by the HPG axis.

## Conclusion

This study revealed the key lncRNAs and mRNAs involved in the HPG axis regulating the reproduction of tongue sole. The enrichment analysis indicated that the target genes of the DE lncRNAs were enriched in a series of pathways related to reproduction. We constructed lncRNA-mRNA regulatory networks based on *antisense*, *cis* and *trans* regulatory mechanisms. In addition, based on the ceRNA hypothesis, we constructed mRNA-miRNA-lncRNA (i.e., ceRNA) networks to predict the target genes of lncRNAs via miRNAs. In summary, this study provides a valuable resource for lncRNAs modulating the expression of mRNAs that play a crucial role in tongue sole reproduction and offers deeper insight into the molecular mechanisms fundamental to improving the efficiency of tongue sole reproduction.

## Data Availability

The datasets presented in this study can be found in online repositories. The names of the repository/repositories and accession number(s) can be found below: https://www.ncbi.nlm.nih.gov/, SRR14090724, SRR14090723, SRR14090712; SRR14090720, SRR14090719, SRR14090718; SRR14090707, SRR14090706, SRR14090705; SRR14090701, SRR14090694, SRR14090693; SRR14090717, SRR14090716, SRR14090715; SRR14090704, SRR14090703, SRR14090702.

## References

[B1] Abdelmohsen K. Panda A. Kang M.-J. Xu J. Selimyan R. Yoon J.-H. (2013). Senescence-associated lncRNAs: Senescence-Associated Long Noncoding RNAs. Aging cell 12, 890–900. 10.1111/acel.12115 23758631 PMC3773026

[B2] Abreu A. P. Kaiser U. B. (2016). Pubertal Development and Regulation. Lancet Diabetes Endocrinol. 4, 254–264. 10.1016/s2213-8587(15)00418-0 26852256 PMC5192018

[B3] Ali A. Al-Tobasei R. Kenney B. Leeds T. D. Salem M. (2018). Integrated Analysis of lncRNA and mRNA Expression in Rainbow trout Families Showing Variation in Muscle Growth and Fillet Quality Traits. Sci. Rep. 8, 12111. 10.1038/s41598-018-30655-8 30108261 PMC6092380

[B4] Ann Sorbera L. Francisco Asturiano J. Carrillo M. Zanuy S. (2001). Effects of Polyunsaturated Fatty Acids and Prostaglandins on Oocyte Maturation in a Marine Teleost, the European Sea Bass (*Dicentrarchus labrax*)1. Biol. Reprod. 64, 382–389. 10.1095/biolreprod64.1.382 11133697

[B5] Arun G. Akhade V. S. Donakonda S. Rao M. R. S. (2012). Mrhl RNA, a Long Noncoding RNA, Negatively Regulates Wnt Signaling through its Protein Partner Ddx5/p68 in Mouse Spermatogonial Cells. Mol. Cel. Biol. 32, 3140–3152. 10.1128/mcb.00006-12 PMC3434522 22665494

[B6] Blanco A. M. (2020). Hypothalamic- and Pituitary-Derived Growth and Reproductive Hormones and the Control of Energy Balance in Fish. Gen. Comp. Endocrinol. 287, 113322. 10.1016/j.ygcen.2019.113322 31738909

[B7] Cai J. Li L. Song L. Xie L. Luo F. Sun S. (2019). Effects of Long Term Antiprogestine Mifepristone (RU486) Exposure on Sexually Dimorphic lncRNA Expression and Gonadal Masculinization in Nile tilapia (*Oreochromis niloticus*). Aquat. Toxicol. 215, 105289. 10.1016/j.aquatox.2019.105289 31491707

[B8] Chang J. P. Pemberton J. G. (2018). Comparative Aspects of GnRH-Stimulated Signal Transduction in the Vertebrate Pituitary - Contributions from Teleost Model Systems. Mol. Cell Endocrinol. 463, 142–167. 10.1016/j.mce.2017.06.002 28587765

[B9] Chen S. Zhang G. Shao C. Huang Q. Liu G. Zhang P. (2014). Whole-genome Sequence of a Flatfish Provides Insights into ZW Sex Chromosome Evolution and Adaptation to a Benthic Lifestyle. Nat. Genet. 46, 253–260. 10.1038/ng.2890 24487278

[B10] Chen S. Zhou Y. Chen Y. Gu J. (2018). Fastp: an Ultra-fast All-In-One FASTQ Preprocessor. Bioinformatics 34, i884–i890. 10.1093/bioinformatics/bty560 30423086 PMC6129281

[B11] Chen Y. G. Satpathy A. T. Chang H. Y. (2017). Gene Regulation in the Immune System by Long Noncoding RNAs. Nat. Immunol. 18, 962–972. 10.1038/ni.3771 28829444 PMC9830650

[B12] Coss D. (2018). Regulation of Reproduction via Tight Control of Gonadotropin Hormone Levels. Mol. Cell Endocrinol. 463, 116–130. 10.1016/j.mce.2017.03.022 28342855 PMC6457911

[B13] Curry T. E. Jr Osteen K. G. (2003). The Matrix Metalloproteinase System: Changes, Regulation, and Impact throughout the Ovarian and Uterine Reproductive Cycle. Endocr. Rev. 24, 428–465. 10.1210/er.2002-0005 12920150

[B14] Deck C. A. Gary Anderson W. Walsh P. J. (2017). Effects of Glucose and Insulin Administration on Glucose Transporter Expression in the North Pacific Spiny Dogfish ( Squalus Suckleyi ). Gen. Comp. Endocrinol. 247, 46–52. 10.1016/j.ygcen.2017.01.016 28093310

[B15] Degani G. Yom-Din S. Goldberg D. Jackson K. (2010). cDNA Cloning of Blue Gourami (*Trichogaster trichopterus*) Prolactin and its Expression during the Gonadal Cycles of Males and Females. J. Endocrinol. Invest. 33, 7–12. 10.1007/bf03346543 20203536

[B16] Dettleff P. Hormazabal E. Aedo J. Fuentes M. Meneses C. Molina A. (2020). Identification and Evaluation of Long Noncoding RNAs in Response to Handling Stress in Red Cusk-Eel (*Genypterus Chilensis*) via RNA-Seq. Mar. Biotechnol. 22, 94–108. 10.1007/s10126-019-09934-6 31748906

[B17] Dobolyi A. Oláh S. Keller D. Kumari R. Fazekas E. A. Csikós V. (2020). Secretion and Function of Pituitary Prolactin in Evolutionary Perspective. Front. Neurosci. 14, 621. 10.3389/fnins.2020.00621 32612510 PMC7308720

[B18] Dong Q. Lazarus R. M. Wong L. S. Vellios M. Handelsman D. J. (1991). Pulsatile LH Secretion in Streptozotocin-Induced Diabetes in the Rat. J. Endocrinol. 131, 49–55. 10.1677/joe.0.1310049 1744558

[B19] Dong Y. Lyu L. Zhang D. Li J. Wen H. Shi B. (2021). Integrated lncRNA and mRNA Transcriptome Analyses in the Ovary of *Cynoglossus Semilaevis* Reveal Genes and Pathways Potentially Involved in Reproduction. Front. Genet. 12, 671729. 10.3389/fgene.2021.671729 34093665 PMC8172126

[B20] Du X. Li Q. Yang L. Liu L. Cao Q. Li Q. (2020). SMAD4 Activates Wnt Signaling Pathway to Inhibit Granulosa Cell Apoptosis. Cell Death Dis 11, 373. 10.1038/s41419-020-2578-x 32415058 PMC7228950

[B21] Dykes I. M. Emanueli C. (2017). Transcriptional and post-transcriptional Gene Regulation by Long Non-coding RNA. Genomics, Proteomics & Bioinformatics 15, 177–186. 10.1016/j.gpb.2016.12.005 PMC5487525 28529100

[B22] Faghihi M. A. Zhang M. Huang J. Modarresi F. Van der Brug M. P. Nalls M. A. (2010). Evidence for Natural Antisense Transcript-Mediated Inhibition of microRNA Function. Genome Biol. 11, R56. 10.1186/gb-2010-11-5-r56 20507594 PMC2898074

[B23] Fatica A. Bozzoni I. (2014). Long Non-coding RNAs: New Players in Cell Differentiation and Development. Nat. Rev. Genet. 15, 7–21. 10.1038/nrg3606 24296535

[B24] Feng B. Li S. Wang Q. Tang L. Huang F. Zhang Z. (2021). lncRNA DMRT2-AS Acts as a Transcriptional Regulator of Dmrt2 Involving in Sex Differentiation in the Chinese Tongue Sole (*Cynoglossus Semilaevis*). Comp. Biochem. Physiol. B: Biochem. Mol. Biol. 253, 110542. 10.1016/j.cbpb.2020.110542 33301875

[B25] Gu W. Yang Y. Ning C. Wang Y. Hu J. Zhang M. (2021). Identification and Characteristics of Insulin-like Growth Factor System in the Brain, Liver, and Gonad during Development of a Seasonal Breeding Teleost, Pampus Argenteus. Gen. Comp. Endocrinol. 300, 113645. 10.1016/j.ygcen.2020.113645 33058908

[B26] Guiguen Y. Fostier A. Piferrer F. Chang C.-F. (2010). Ovarian Aromatase and Estrogens: a Pivotal Role for Gonadal Sex Differentiation and Sex Change in Fish. Gen. Comp. Endocrinol. 165, 352–366. 10.1016/j.ygcen.2009.03.002 19289125

[B27] Hileman S. M. Schillo K. K. Hall J. B. (1993). Effects of Acute, Intracerebroventricular Administration of Insulin on Serum Concentrations of Luteinizing Hormone, Insulin, and Glucose in Ovariectomized Lambs during Restricted and Ad Libitum Feed Intake1. Biol. Reprod. 48, 117–124. 10.1095/biolreprod48.1.117 8418899

[B28] Hombach S. Kretz M. (2016). Non-coding RNAs: Classification, Biology and Functioning. Adv. Exp. Med. Biol. 937, 3–17. 10.1007/978-3-319-42059-2_1 27573892

[B29] Hrabia A. Wolak D. Sechman A. (2021). Response of the Matrix Metalloproteinase System of the Chicken Ovary to Prolactin Treatment. Theriogenology 169, 21–28. 10.1016/j.theriogenology.2021.04.002 33915314

[B30] Hu Q. Qin Q. Xu S. Zhou L. Xia C. Shi X. (2020). Pituitary Actions of EGF on Gonadotropins, Growth Hormone, Prolactin and Somatolactins in Grass Carp. Biology 9, 279. 10.3390/biology9090279 PMC7564354 32911654

[B31] Imaoka T. Matsuda M. Mori T. (2000). Extrapituitary Expression of the Prolactin Gene in the Goldfish, African Clawed Frog and Mouse. Zoolog. Sci. 17, 791–796. 10.2108/zsj.17.791

[B32] Janjic M. M. Prévide R. M. Fletcher P. A. Sherman A. Smiljanic K. Abebe D. (201920098). Divergent Expression Patterns of Pituitary Gonadotropin Subunit and GnRH Receptor Genes to Continuous GnRH *In Vitro* and *In Vivo* . Sci. Rep. 9. 10.1038/s41598-019-56480-1 PMC6934515 31882740

[B33] Kim D. Pertea G. Trapnell C. Pimentel H. Kelley R. Salzberg S. L. (2013). TopHat2: Accurate Alignment of Transcriptomes in the Presence of Insertions, Deletions and Gene Fusions. Genome Biol. 14, R36. 10.1186/gb-2013-14-4-r36 23618408 PMC4053844

[B34] Kong L. Zhang Y. Ye Z.-Q. Liu X.-Q. Zhao S.-Q. Wei L. (2007). CPC: Assess the Protein-Coding Potential of Transcripts Using Sequence Features and Support Vector Machine. Nucleic Acids Res. 35, W345–w349. 10.1093/nar/gkm391 17631615 PMC1933232

[B35] Kopp F. Mendell J. T. (2018). Functional Classification and Experimental Dissection of Long Noncoding RNAs. Cell 172, 393–407. 10.1016/j.cell.2018.01.011 29373828 PMC5978744

[B36] Lang C. Dai Y. Wu Z. Yang Q. He S. Zhang X. (2020). SMAD3/SP1 Complex‐mediated Constitutive Active Loop between lncRNA PCAT7 and TGF‐β Signaling Promotes Prostate Cancer Bone Metastasis. Mol. Oncol. 14, 808–828. 10.1002/1878-0261.12634 31925912 PMC7138406

[B37] Langmead B. Salzberg S. L. (2012). Fast Gapped-Read Alignment with Bowtie 2. Nat. Methods 9, 357–359. 10.1038/nmeth.1923 22388286 PMC3322381

[B38] Lau M.-T. Ge W. (2005). Cloning of Smad2, Smad3, Smad4, and Smad7 from the Goldfish Pituitary and Evidence for Their Involvement in Activin Regulation of Goldfish FSHβ Promoter Activity. Gen. Comp. Endocrinol. 141, 22–38. 10.1016/j.ygcen.2004.10.019 15707600

[B39] Li B. Dewey C. N. (2011). RSEM: Accurate Transcript Quantification from RNA-Seq Data with or without a Reference Genome. BMC Bioinformatics 12, 323. 10.1186/1471-2105-12-323 21816040 PMC3163565

[B40] Lian Z. Zou X. Han Y. Deng M. Sun B. Guo Y. (2020). Role of mRNAs and Long Non‐coding RNAs in Regulating the Litter Size Trait in Chuanzhong Black Goats. Reprod. Dom Anim. 55, 486–495. 10.1111/rda.13642 31960497

[B41] Lin C.-J. Maugars G. Lafont A.-G. Jeng S.-R. Wu G.-C. Dufour S. (2020). Basal Teleosts Provide New Insights into the Evolutionary History of Teleost-Duplicated Aromatase. Gen. Comp. Endocrinol. 291, 113395. 10.1016/j.ygcen.2020.113395 31981691

[B42] Lin S.-W. Ge W. (2009). Differential Regulation of Gonadotropins (FSH and LH) and Growth Hormone (GH) by Neuroendocrine, Endocrine, and Paracrine Factors in the Zebrafish-An *In Vitro* Approach. Gen. Comp. Endocrinol. 160, 183–193. 10.1016/j.ygcen.2008.11.020 19063890

[B43] Liu J. Du X. Zhou J. Pan Z. Liu H. Li Q. (2014). MicroRNA-26b Functions as a Proapoptotic Factor in Porcine Follicular Granulosa Cells by Targeting Sma-And Mad-Related Protein 41. Biol. Reprod. 91, 146. 10.1095/biolreprod.114.122788 25395673

[B44] Liu K. Ji F. Yang G. Hou Z. Sun J. Wang X. (2016). SMAD4 Defect Causes Auditory Neuropathy via Specialized Disruption of Cochlear Ribbon Synapses in Mice. Mol. Neurobiol. 53, 5679–5691. 10.1007/s12035-015-9454-1 26491026

[B45] Liu X. Li W. Jiang L. Lü Z. Liu M. Gong L. (2019). Immunity-associated Long Non-coding RNA and Expression in Response to Bacterial Infection in Large Yellow Croaker (*Larimichthys Crocea*). Fish Shellfish Immunol. 94, 634–642. 10.1016/j.fsi.2019.09.015 31533082

[B46] Livak K. J. Schmittgen T. D. (2001). Analysis of Relative Gene Expression Data Using Real-Time Quantitative PCR and the 2−ΔΔCT Method. Methods 25, 402–408. 10.1006/meth.2001.1262 11846609

[B47] Lynn S. G. Shepherd B. S. (2007). Molecular Characterization and Sex-specific Tissue Expression of Prolactin, Somatolactin and Insulin-like Growth Factor-I in Yellow Perch (*Perca flavescens*). Comp. Biochem. Physiol. Part B: Biochem. Mol. Biol. 147, 412–427. 10.1016/j.cbpb.2007.02.005 17418604

[B48] Mahardini A. Yamauchi C. Takeuchi Y. Rizky D. Takekata H. Takemura A. (2018). Changes in mRNA Abundance of Insulin-like Growth Factors in the Brain and Liver of a Tropical Damselfish, *Chrysiptera Cyanea*, in Relation to Seasonal and Food-Manipulated Reproduction. Gen. Comp. Endocrinol. 269, 112–121. 10.1016/j.ygcen.2018.09.001 30189192

[B49] Mercer T. R. Mattick J. S. (2013). Structure and Function of Long Noncoding RNAs in Epigenetic Regulation. Nat. Struct. Mol. Biol. 20, 300–307. 10.1038/nsmb.2480 23463315

[B50] M. Irwin D. (2019). Duplication and Diversification of Insulin Genes in ray-finned Fish. Zool Res. 40, 185–197. 10.24272/j.issn.2095-8137.2018.052 30127332 PMC6591164

[B51] Ogiwara K. Takano N. Shinohara M. Murakami M. Takahashi T. (2005). Gelatinase A and Membrane-type Matrix Metalloproteinases 1 and 2 Are Responsible for Follicle Rupture during Ovulation in the Medaka. Proc. Natl. Acad. Sci. 102, 8442–8447. 10.1073/pnas.0502423102 15941829 PMC1150835

[B52] Przylipiak A. Kiesel L. Rabe T. Helm K. Przylipiak M. Runnebaum B. (1988). Epidermal Growth Factor Stimulates Luteinizing Hormone and Arachidonic Acid Release in Rat Pituitary Cells. Mol. Cell Endocrinol. 57, 157–162. 10.1016/0303-7207(88)90045-7 3260875

[B53] Quan J. Kang Y. Luo Z. Zhao G. Ma F. Li L. (2020). Identification and Characterization of Long Noncoding RNAs Provide Insight into the Regulation of Gene Expression in Response to Heat Stress in Rainbow trout (*Oncorhynchus mykiss*). Comp. Biochem. Physiol. D: Genomics Proteomics 36, 100707. 10.1016/j.cbd.2020.100707 32693384

[B54] Quinn J. J. Chang H. Y. (2016). Unique Features of Long Non-coding RNA Biogenesis and Function. Nat. Rev. Genet. 17, 47–62. 10.1038/nrg.2015.10 26666209

[B55] Qureshi I. A. Mattick J. S. Mehler M. F. (2010). Long Non-coding RNAs in Nervous System Function and Disease. Brain Res. 1338, 20–35. 10.1016/j.brainres.2010.03.110 20380817 PMC2883659

[B56] Roelle S. Grosse R. Aigner A. Krell H. W. Czubayko F. Gudermann T. (2003). Matrix Metalloproteinases 2 and 9 Mediate Epidermal Growth Factor Receptor Transactivation by Gonadotropin-Releasing Hormone. J. Biol. Chem. 278, 47307–47318. 10.1074/jbc.M304377200 12963732

[B57] Salem O. Erdem N. Jung J. Münstermann E. Wörner A. Wilhelm H. (2016). The Highly Expressed 5'isomiR of Hsa-miR-140-3p Contributes to the Tumor-Suppressive Effects of miR-140 by Reducing Breast Cancer Proliferation and Migration. BMC Genomics 17, 566. 10.1186/s12864-016-2869-x 27502506 PMC4977694

[B58] Shah B. H. Catt K. J. (2004). Matrix Metalloproteinases in Reproductive Endocrinology. Trends Endocrinol. Metab. 15, 47–49. 10.1016/j.tem.2004.01.004 15080147

[B59] Shi B. Liu X. Thomas P. Pang Y. Xu Y. Li X. (2016). Identification and Characterization of a Progestin and adipoQ Receptor (PAQR) Structurally Related to Paqr7 in the Ovary of *Cynoglossus Semilaevis* and its Potential Role in Regulating Oocyte Maturation. Gen. Comp. Endocrinol. 237, 109–120. 10.1016/j.ygcen.2016.08.008 27554928

[B60] Shi B. Liu X. Xu Y. Wang S. (2015). Molecular Characterization of Three Gonadotropin Subunits and Their Expression Patterns during Ovarian Maturation in *Cynoglossus Semilaevis* . Ijms 16, 2767–2793. 10.3390/ijms16022767 25633101 PMC4346864

[B61] Sîrbulescu R. F. Ilieş I. Zupanc G. K. H. (2015). Matrix Metalloproteinase-2 and -9 in the Cerebellum of Teleost Fish: Functional Implications for Adult Neurogenesis. Mol. Cell Neurosci. 68, 9–23. 10.1016/j.mcn.2015.03.015 25827096

[B62] Sliwowska J. H. Fergani C. Gawałek M. Skowronska B. Fichna P. Lehman M. N. (2014). Insulin: its Role in the central Control of Reproduction. Physiol. Behav. 133, 197–206. 10.1016/j.physbeh.2014.05.021 24874777 PMC4084551

[B63] Smith P. C. Martínez C. Cáceres M. Martínez J. (20152000). Research on Growth Factors in Periodontology. Periodontol. 2000 67, 234–250. 10.1111/prd.12068 25494603

[B64] Song Y. Zheng W. Zhang M. Cheng X. Cheng J. Wang W. (2020). Out-of-season Artificial Reproduction Techniques of Cultured Female Tongue Sole (*Cynoglossus Semilaevis*): Broodstock Management, Administration Methods of Hormone Therapy and Artificial Fertilization. Aquaculture 518, 734866. 10.1016/j.aquaculture.2019.734866

[B65] Sorokowski P. Żelaźniewicz A. Nowak J. Groyecka A. Kaleta M. Lech W. (2019). Romantic Love and Reproductive Hormones in Women. Ijerph 16, 4224. 10.3390/ijerph16214224 PMC6861983 31683520

[B66] Sun L. Luo H. Bu D. Zhao G. Yu K. Zhang C. (2013). Utilizing Sequence Intrinsic Composition to Classify Protein-Coding and Long Non-coding Transcripts. Nucleic Acids Res. 41, e166. 10.1093/nar/gkt646 23892401 PMC3783192

[B67] Takahashi T. Fujimori C. Hagiwara A. Ogiwara K. (2013). Recent Advances in the Understanding of Teleost Medaka Ovulation: the Roles of Proteases and Prostaglandins. Zoolog. Sci. 30, 239–247. 10.2108/zsj.30.239 23537233

[B68] Trapnell C. Roberts A. Goff L. Pertea G. Kim D. Kelley D. R. (2012). Differential Gene and Transcript Expression Analysis of RNA-Seq Experiments with TopHat and Cufflinks. Nat. Protoc. 7, 562–578. 10.1038/nprot.2012.016 22383036 PMC3334321

[B69] Tsai M.-C. Manor O. Wan Y. Mosammaparast N. Wang J. K. Lan F. (2010). Long Noncoding RNA as Modular Scaffold of Histone Modification Complexes. Science 329, 689–693. 10.1126/science.1192002 20616235 PMC2967777

[B70] Ulhaq Z. S. Kishida M. (2018). Brain Aromatase Modulates Serotonergic Neuron by Regulating Serotonin Levels in Zebrafish Embryos and Larvae. Front. Endocrinol. 9, 230. 10.3389/fendo.2018.00230 PMC5954033 29867763

[B71] Vila-Porcile E. Picart R. Vigny M. Tixier-Vidal A. Tougard C. (1992). Immunolocalization of Laminin, Heparan-Sulfate Proteoglycan, Entactin, and Type IV Collagen in the Rat Anterior Pituitary. I. An *In Vivo* Study. Anat. Rec. 232, 482–492. 10.1002/ar.1092320405 1554101

[B72] Walker C. Mojares E. del Río Hernández A. (2018). Role of Extracellular Matrix in Development and Cancer Progression. Ijms 19, 3028. 10.3390/ijms19103028 PMC6213383 30287763

[B73] Whittington C. M. Wilson A. B. (2013). The Role of Prolactin in Fish Reproduction. Gen. Comp. Endocrinol. 191, 123–136. 10.1016/j.ygcen.2013.05.027 23791758

[B74] Williamson C. M. Ball S. T. Dawson C. Mehta S. Beechey C. V. Fray M. (2011). Uncoupling Antisense-Mediated Silencing and DNA Methylation in the Imprinted Gnas Cluster. Plos Genet. 7, e1001347. 10.1371/journal.pgen.1001347 21455290 PMC3063750

[B75] Wu S. Zhang J. Liu B. Huang Y. Li S. Wen H. (2020). Identification and Characterization of lncRNAs Related to the Muscle Growth and Development of Japanese Flounder (*Paralichthys olivaceus*). Front. Genet. 11, 1034. 10.3389/fgene.2020.01034 33033494 PMC7510837

[B76] Yamada H. Kimura T. Yamada H. (2019). Regulatory Non-coding RNAs in Nervous System Development and Disease. Front. Biosci. 24, 1203–1240. 10.2741/4776 31136976

[B77] Yang B.-Y. Greene M. Chen T. T. (1999). Early Embryonic Expression of the Growth Hormone Family Protein Genes in the Developing Rainbow trout,*Oncorhynchus mykiss* . Mol. Reprod. Dev. 53, 127–134. 10.1002/(sici)1098-2795(199906)53:2<127:aid-mrd1>3.0.co;2-h 10331450

[B78] Yang C. X. Wang P. C. Liu S. Miao J. K. Liu X. M. Miao Y. L. (2020). Long Noncoding RNA 2193 Regulates Meiosis through Global Epigenetic Modification and Cytoskeleton Organization in Pig Oocytes. J. Cel Physiol 235, 8304–8318. 10.1002/jcp.29675 32239703

[B79] Yang L. Lin C. Jin C. Yang J. C. Tanasa B. Li W. (2013). lncRNA-Dependent Mechanisms of Androgen-Receptor-Regulated Gene Activation Programs. Nature 500, 598–602. 10.1038/nature12451 23945587 PMC4034386

[B80] Yaron Z. Levavi-Sivan B. (2011). “Hormonal Control of Reproduction and Growth | Endocrine Regulation of Fish Reproduction,” in Endocrine Regulation of Fish reproductionEncyclopedia of Fish Physiology: From Genome to Environment. Editor Farrell A. P. (San Diego, CA, USA: Academic Press), 1500–1508. 10.1016/b978-0-12-374553-8.00058-7

[B81] Yu C. Zhang Y.-L. Fan H.-Y. (2013). Selective Smad4 Knockout in Ovarian Preovulatory Follicles Results in Multiple Defects in Ovulation. Mol. Endocrinol. 27, 966–978. 10.1210/me.2012-1364 23592428 PMC5415278

[B82] Zhang C. Hao Y. Wang Y. Xu J. Teng Y. Yang X. (2018). TGF-β/SMAD4-Regulated LncRNA-LINP1 Inhibits Epithelial-Mesenchymal Transition in Lung Cancer. Int. J. Biol. Sci. 14, 1715–1723. 10.7150/ijbs.27197 30416386 PMC6216035

[B83] Zhang W. Tian J. Zhang L. Zhang Y. Li X. Lin H. (2004). cDNA Sequence and Spatio-Temporal Expression of Prolactin in the orange-spotted Grouper, *Epinephelus coioides* . Gen. Comp. Endocrinol. 136, 134–142. 10.1016/j.ygcen.2003.12.001 14980804

[B84] Zhang Y.-y. Tang P. M.-K. Tang P. C.-T. Xiao J. Huang X.-R. Yu C. (2019). LRNA9884, a Novel Smad3-dependent Long Noncoding RNA, Promotes Diabetic Kidney Injury in *Db/db* Mice via Enhancing MCP-1-dependent Renal Inflammation. Diabetes 68, 1485–1498. 10.2337/db18-1075 31048367

[B85] Zheng W. Chu Q. Xu T. (2021). Long Noncoding RNA IRL Regulates NF-Κb-Mediated Immune Responses through Suppression of miR-27c-3p-dependent IRAK4 Downregulation in Teleost Fish. J. Biol. Chem. 296, 100304. 10.1016/j.jbc.2021.100304 33465375 PMC7949060

[B86] Zhuang J. Shen L. Yang L. Huang X. Lu Q. Cui Y. (2017). TGFβ1 Promotes Gemcitabine Resistance through Regulating the LncRNA-LET/NF90/miR-145 Signaling Axis in Bladder Cancer. Theranostics 7, 3053–3067. 10.7150/thno.19542 28839463 PMC5566105

