# Supplementary material for: Brain and Pituitary Transcriptome Analyses Reveal the Differential Regulation of Reproduction-Related LncRNAs and mRNAs in Cynoglossus semilaevis
Source: Front Genet. 2021 Dec 9;12:802953. doi: 10.3389/fgene.2021.802953 (PMC8696122; doi:10.3389/fgene.2021.802953)
Supplement: Supplementary file 1 [file Table1.DOCX]

Supplementary Material

# Supplementary Figures and Tables

## Supplementary Figures


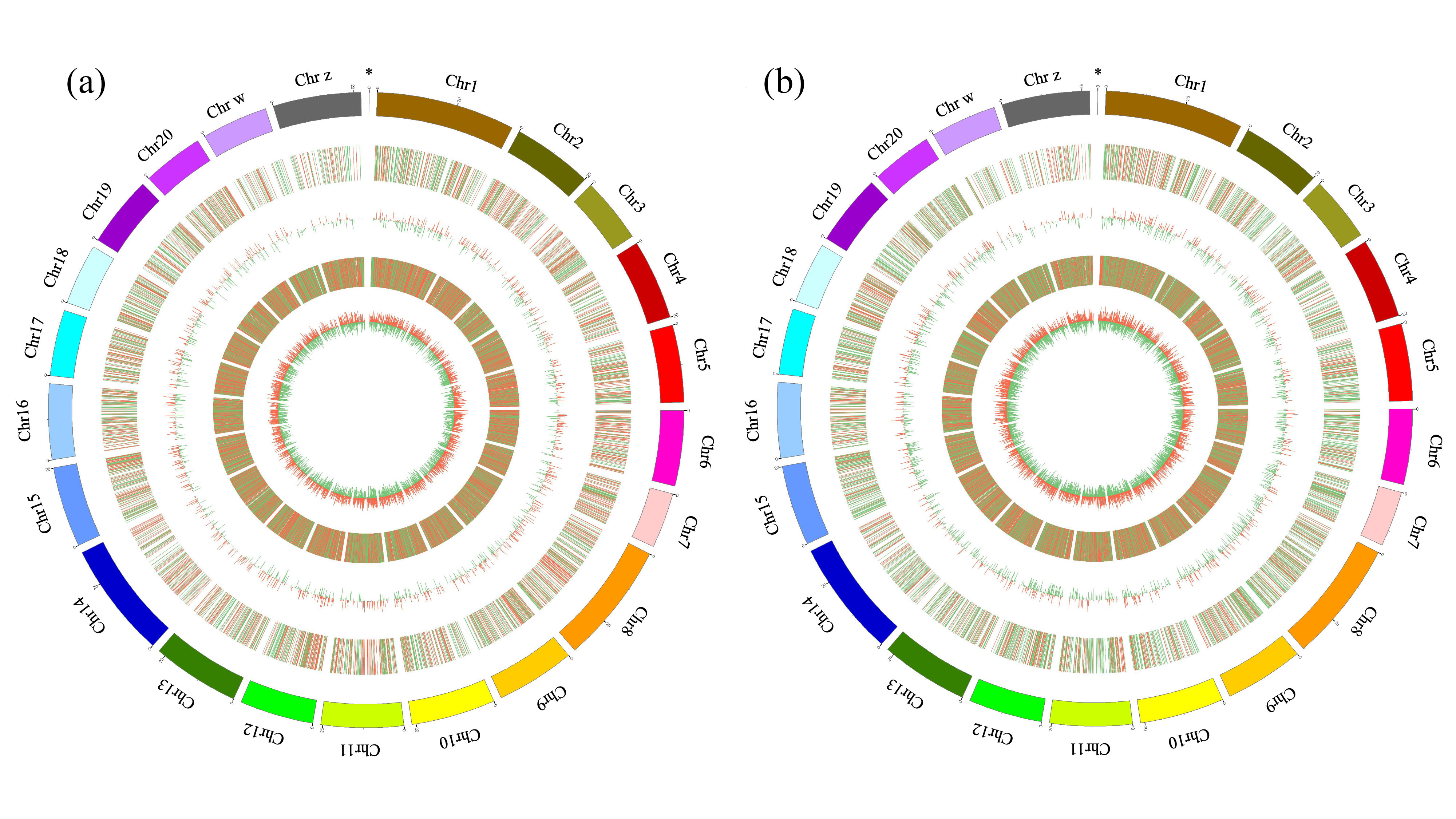


**Supplementary Figure 1**. Circos plot of DE lncRNAs and mRNAs in the pituitary in the stage IV vs. V **(a)** and stage V vs. VI **(b)** comparison groups. The outermost circle shows the autosomal distribution in tongue sole. The second circle shows the chromosomal locations of the DE lncRNAs; the red line represents upregulation, and the green line represents downregulation. The third circle shows the histogram of DE lncRNAs at different positions; red represents upregulation, green represents downregulation, and higher columns indicate higher numbers of DE genes. The fourth circle shows the distribution of DE mRNAs on the chromosomes; the color code is the same as that used to represent lncRNAs. The innermost circle shows the histogram of DE mRNAs at different positions; the color code is the same as that used to represent lncRNAs. The“*” symbol indicates the mitochondrial genome.


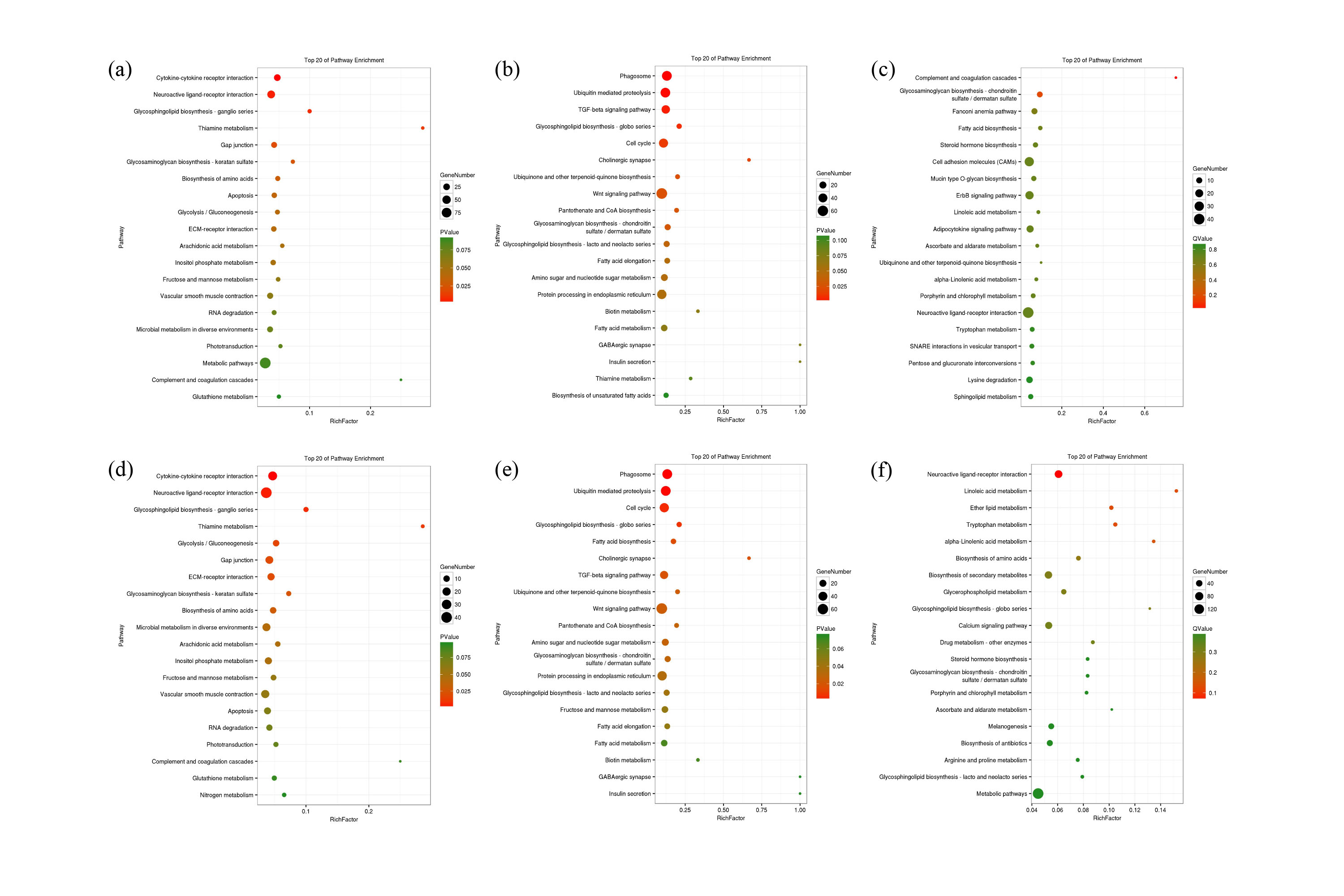


**Supplementary Figure 2**. Top 20 significantly enriched pathways associated with *antisense* (**a**), *cis* (**b**), and *trans* (**c**) target genes in brain; Top 20 significantly enriched pathways associated with *antisense* (**d**), *cis* (**e**), and *trans* (**f**) target genes in pituitary.


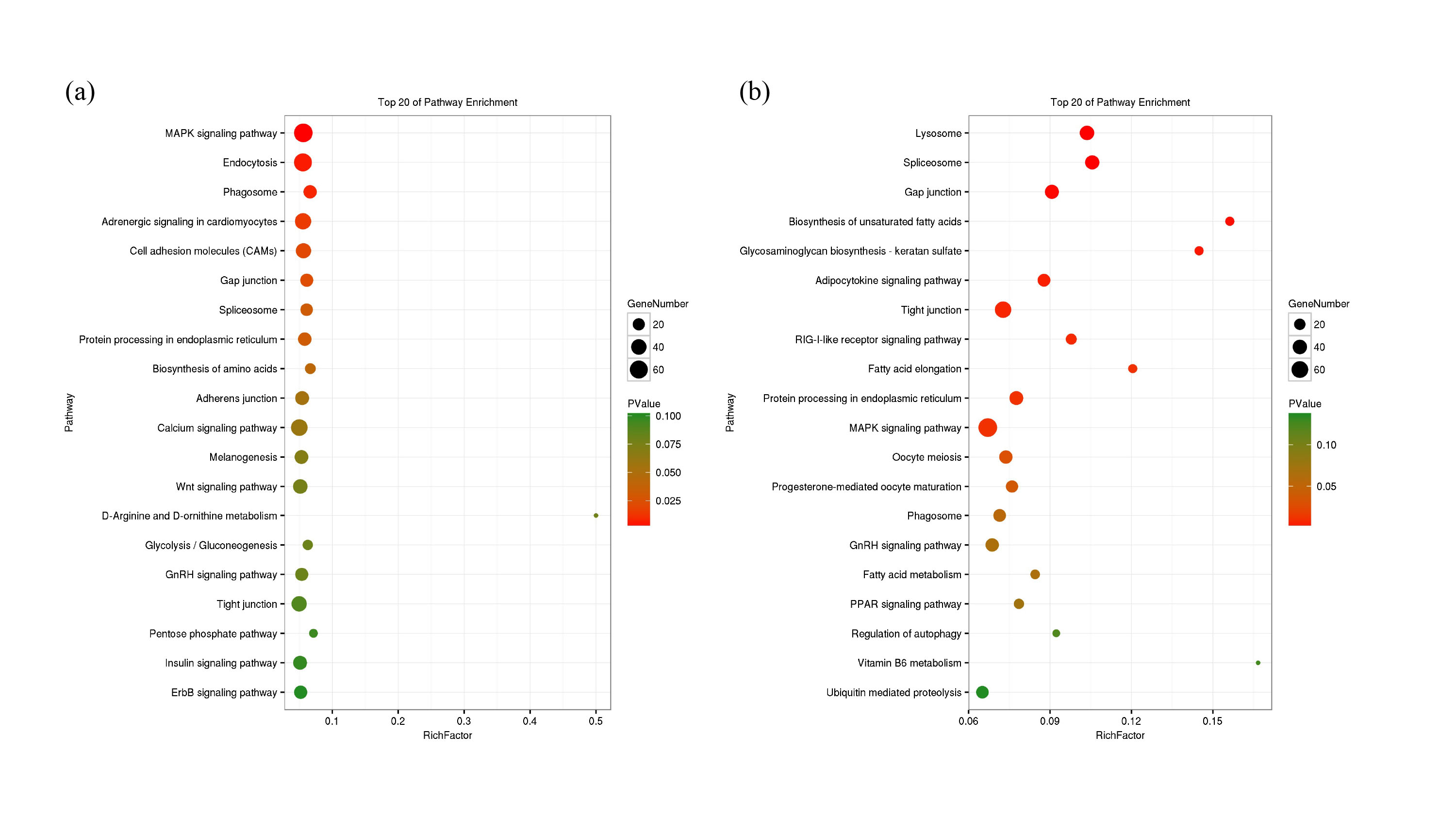


**Supplementary Figure 3**. Top 20 significantly enriched pathways associated with genes in ceRNA network in brain (**a**) and pituitary (**b**).

## Supplementary Tables

**Table S1**. Primer sequences.

**Table S2**. Mapping statistics.

**Table S3**. Identified transcript information.

**Table S4**. Brain differently expressed transcripts.

**Table S5**. Pituitary differently expressed transcripts.

**Table S6**. Significantly enriched GO and KEGG DE mRNA.

**Table S7**. GSEA significantly enriched GO and KEGG.

**Table S8**. DE genes potentially associated with reproduction.

**Table S9**. Significantly enriched GO and KEGG of *antisense*, *cis* and *trans* target genes.

**Table S10**. The interaction of lncRNA and mRNA in ceRNA.

**Table S11**. Significantly enriched GO and KEGG in ceRNA.
