# Supplementary figures and images for: Brain and Pituitary Transcriptome Analyses Reveal the Differential Regulation of Reproduction-Related LncRNAs and mRNAs in Cynoglossus semilaevis
Source: Front Genet. 2021 Dec 9;12:802953. doi: 10.3389/fgene.2021.802953 (PMC8696122; doi:10.3389/fgene.2021.802953)

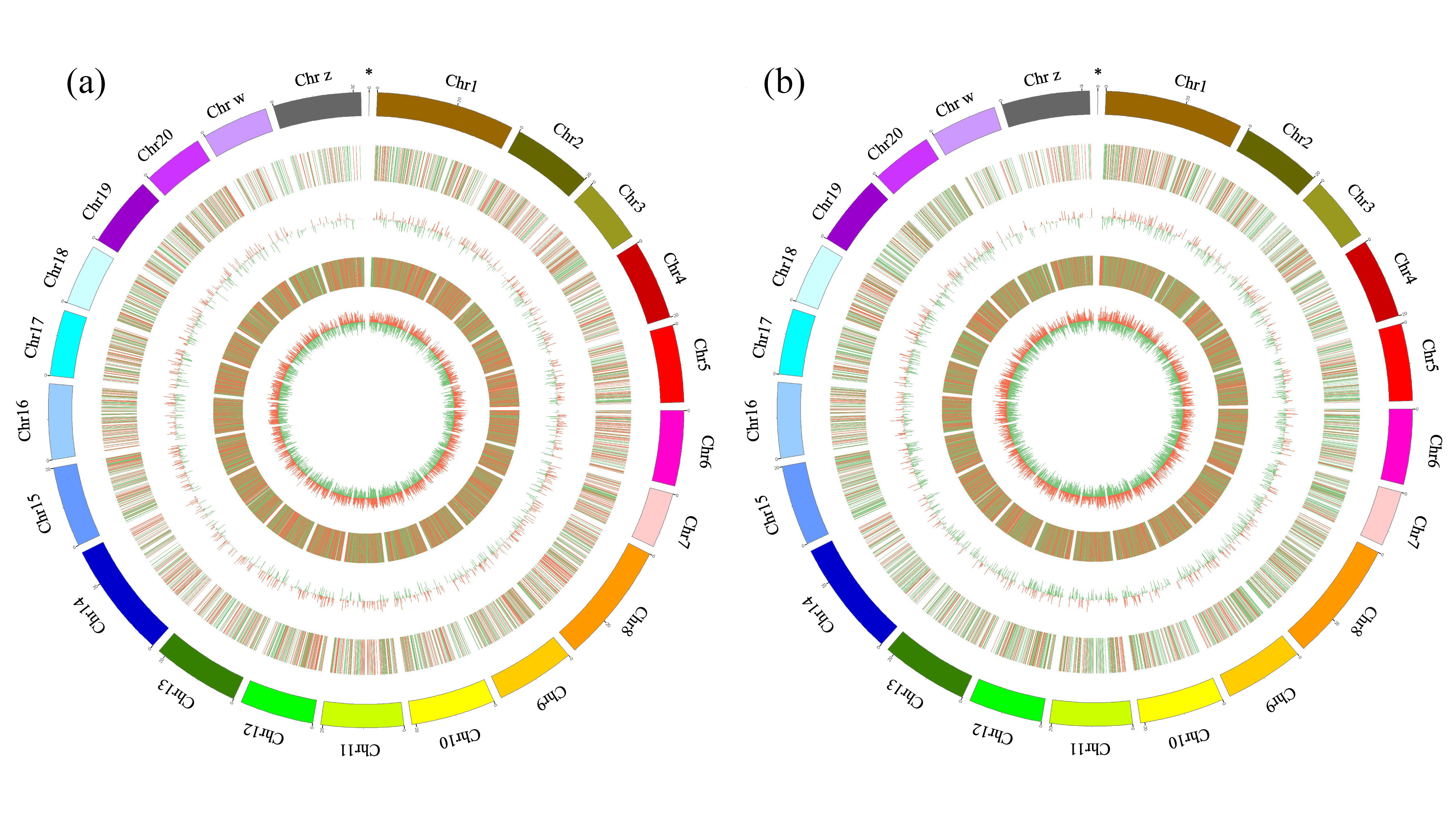

Supplement: Supplementary file 2 [file DataSheet1.ZIP › Supplementary Figures/Supplyment Figure1.jpg]

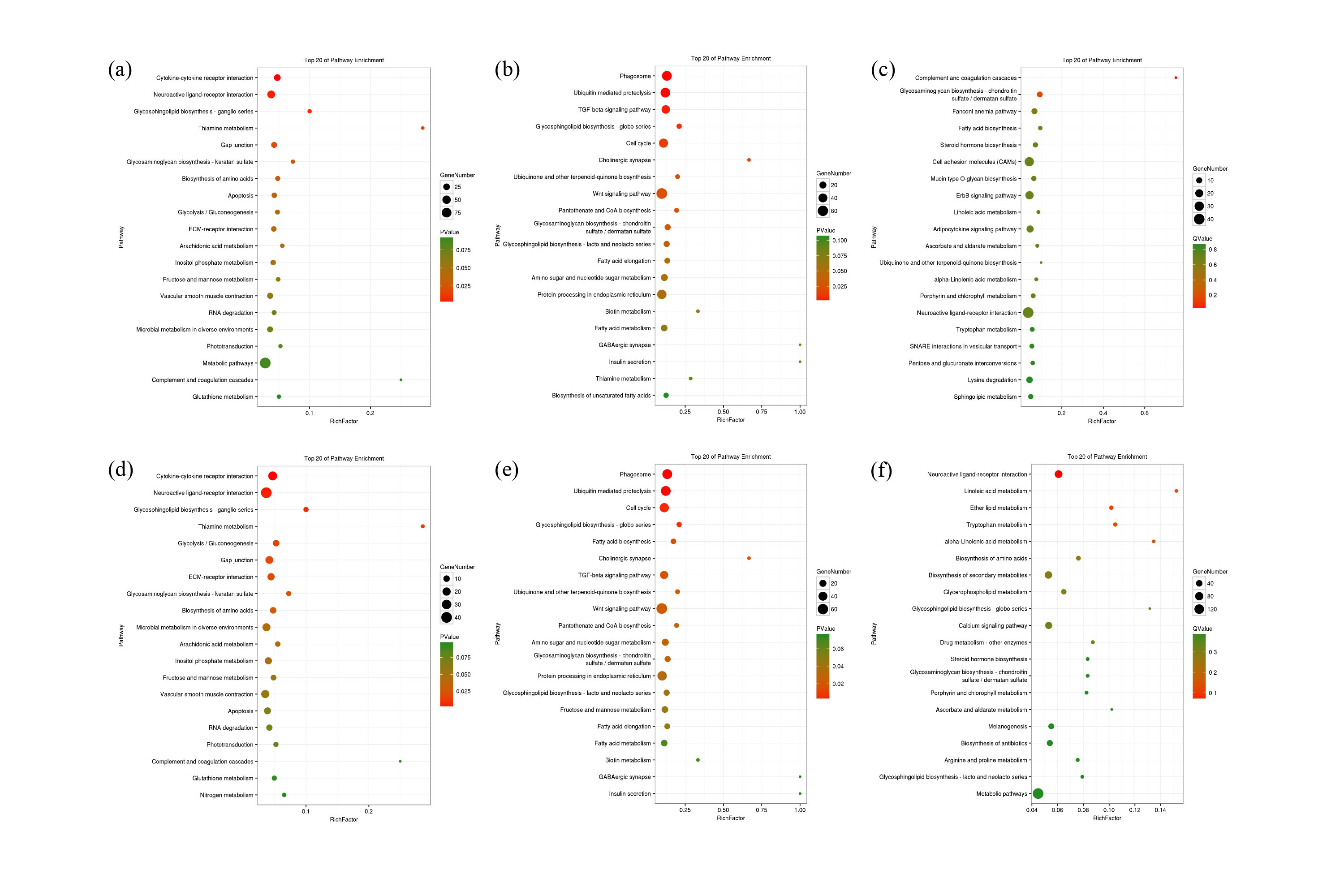

Supplement: Supplementary file 2 [file DataSheet1.ZIP › Supplementary Figures/Supplyment Figure2.jpg]

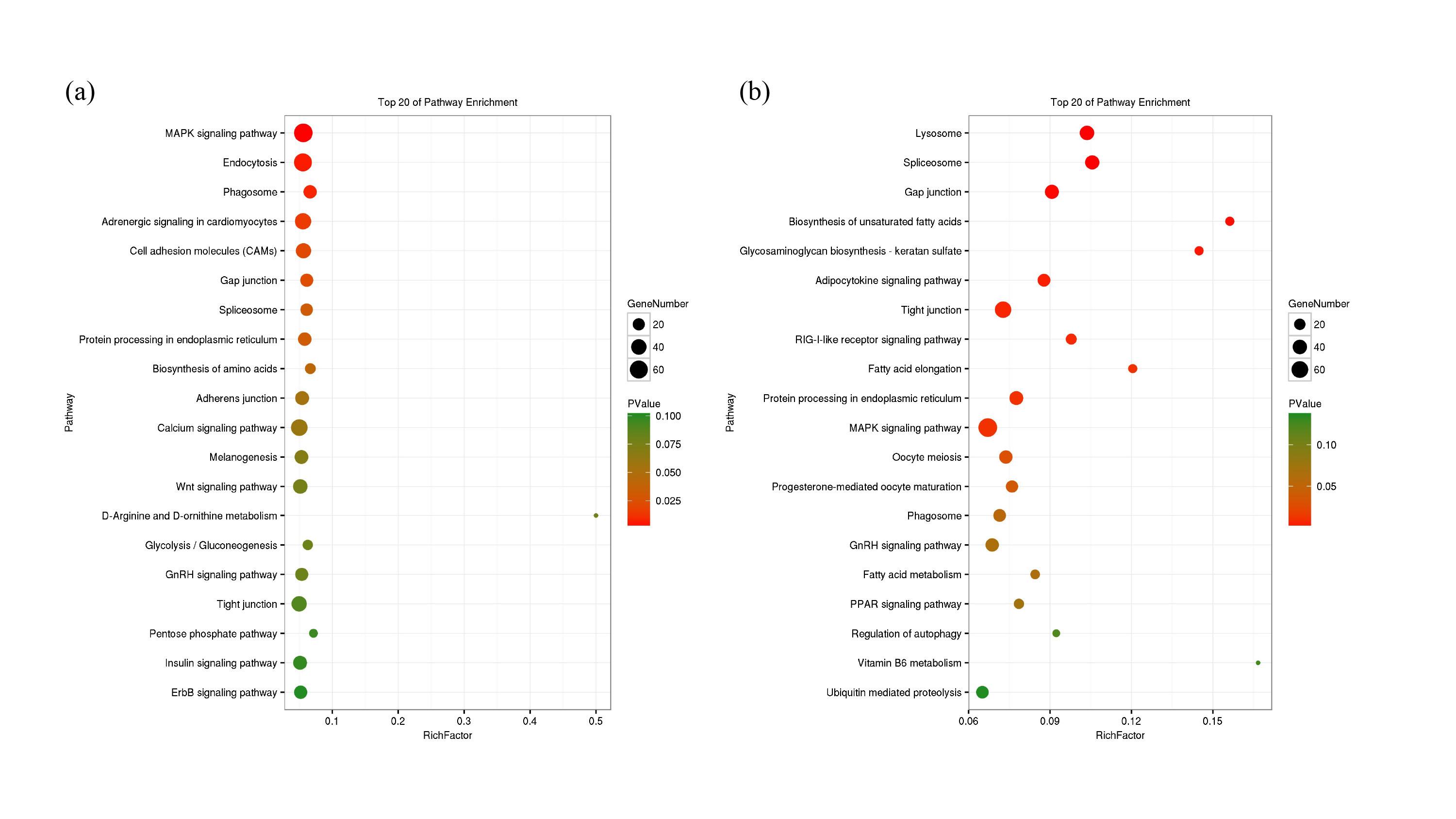

Supplement: Supplementary file 2 [file DataSheet1.ZIP › Supplementary Figures/Supplyment Figure3.jpg]
